# Polydopamine Decorated Microneedles with Fe‐MSC‐Derived Nanovesicles Encapsulation for Wound Healing

**DOI:** 10.1002/advs.202103317

**Published:** 2022-03-10

**Authors:** Wenjuan Ma, Xiaoxuan Zhang, Yuxiao Liu, Lu Fan, Jingjing Gan, Weilin Liu, Yuanjin Zhao, Lingyun Sun

**Affiliations:** ^1^ Department of Rheumatology and Immunology China Pharmaceutical University Nanjing Drum Tower Hospital Nanjing 210008 China; ^2^ Department of Rheumatology and Immunology The Affiliated Drum Tower Hospital of Nanjing University Medical School Nanjing 210008 China; ^3^ State Key Laboratory of Bioelectronics School of Biological Science and Medical Engineering Southeast University Nanjing 210096 China

**Keywords:** hydrogel, microneedle, mesenchymal stem cell, nanovesicle, polydopamine nanoparticles, wound healing

## Abstract

Wound dressing with the capacities of antioxidation, antiinflammation, and efficient angiogenesis induction is expected for effectively promoting wound healing. Herein, a novel core‐shell hyaluronic acid (HA) microneedle (MN) patch with ferrum‐mesenchymal stem cell‐derived artificial nanovesicles (Fe‐MSC‐NVs) and polydopamine nanoparticles (PDA NPs) encapsulated in the needle tips is presented for wound healing. Fe‐MSC‐NVs containing multifunctional therapeutic cytokines are encapsulated in the inner HA core of the MN tips for accelerating angiogenesis. The PDA NPs are encapsulated in the outer methacrylated hyaluronic acid (HAMA) shell of the MN tips to overcome the adverse impacts from reactive oxygen species (ROS)‐derived oxidative stress. With the gradual degradation of HAMA patch tips in the skin, the PDA NPs are sustainably released at the lesion to suppress the ROS‐induced inflammation reaction, while the Fe‐MSC‐NVs significantly increase the migration, proliferation, and tube formation of human umbilical vein endothelial cells (HUVEC). More attractively, the combination of PDA NPs and Fe‐MSC‐NVs further promotes M2 macrophage polarization, thereby suppressing wound inflammation. Through in vivo experiment, the Fe‐MSC‐NVs/PDA MN patch shows an excellent effect for diabetic wound healing. These features of antioxidation, antiinflammation, and pro‐angiogenesis indicate the proposed composite core‐shell MN patch is valuable for clinical wound healing applications.

## Graphical abstract

Core‐shell microneedle (MN) patches with ferrum‐mesenchymal stem cell‐derived nanovesicles (Fe‐MSC‐NVs) and polydopamine nanoparticles (PDA NPs) encapsulated in different layers of tips are developed for promoting wound healing. Their desired features of antioxidation, anti‐inflammation, and pro‐angiogenesis indicate that the proposed composite core‐shell MN patches are valuable for comprehensively handling the complicated process in diabetic wound healing.

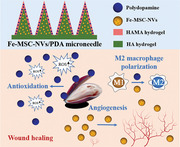

## 1 Introduction

Skin ulcers are the most common cause of diabetes‐associated amputations, which have brought severe problems to patients.^[^ ^1^ ^]^ Generally, these diabetic skin ulcers can be induced by chronic diabetic wounds, which are characterized by impaired angiogenesis, overexpressed reactive oxygen species (ROS), and persistent inflammation.^[^ ^2^ ^]^ Impaired angiogenesis leads to a lack of nutrition and oxygen at the wound sites, inducing persistent inflammation and decreasing collagen production.^[^ ^3^ ^]^ The overexpressed ROS will result in oxidative stress, which causes lipids, proteins, and DNA damage in cells, thus inducing cell death and triggering deleterious processes such as necrosis, inflammation, and fibrotic scarring.^[^ ^4^, ^5^ ^]^ Since the critical role of the impaired angiogenesis and overexpressed ROS,^[^ ^6^ ^]^ the attempts to promote diabetic wound healing have focused on the development of an effective treatment approach based on multifunctional biomaterials,^[^ ^7^, ^8^, ^9^, ^10^ ^]^ which could simultaneously attenuate the oxidative stress, as well as promote angiogenesis at the wound sites.^[^ ^11^ ^]^ Although with many successes, most of the existing biomaterials are merely encapsulating growth factors for inducing angiogenesis or delivering antioxidants for scavenging the ROS overexpression, which is still limited in comprehensively handling the complicated process in wound healing. Therefore, the development of new biomaterial‐derived tools with multiple functions for diabetic wound healing is still anticipated.

**Figure 1:**
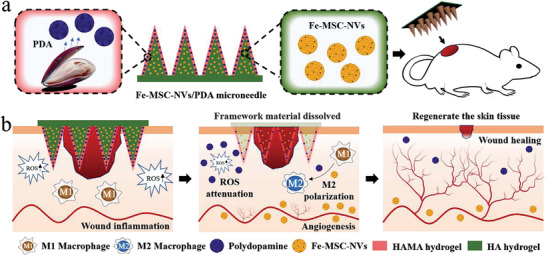
Schematic illustrations of Fe‐MSC‐NVs/PDA MN patch for diabetic wound healing: (a) schematic of Fe‐MSC‐NVs/PDA MN patch; (b) schematic of the wound closure process.

In this paper, we present a novel core‐shell microneedle (MN) patch with mesenchymal stem cell (MSC)‐derived artificial nanovesicles (NVs) and polydopamine nanoparticles (PDA NPs) encapsulated in the needle tips for the wound healing with desired features, as schemed in **Figure** **1**. Compared with conventional hypodermic needles, MN patches can bypass skin's barrier to effectively deliver macromolecules through the microscale needle arrays with limited pain.^[^ ^12^, ^13^, ^14^ ^]^ Also, the designed MN patch could be integrally degradable and biocompatible, which made the drug delivery and absorption more efficient, convenient, and safer.^[^ ^15^, ^16^, ^17^ ^]^ However, traditional MNs often only have a single function, lacking the overall consideration of the complexity of biological and physiological processes. On the other hand, because of their nanoscale diameters and advanced functions inherited from the parent stem cells, MSC‐derived exosomes have provided an effective approach to wound healing.^[^ ^18^, ^19^ ^]^ As a kind of artificial exosomes produced by extruding MSCs through porous membranes filtration steps,^[^ ^20^, ^21^ ^]^ MSC NVs enable a 250‐fold increase in yield production as well as substantially enhanced expression of mRNAs and proteins compared to naturally secreted exosomes,^[^ ^22^, ^23^ ^]^ which indicate much practical clinical values. Although with many successes, the expression amount and level of the therapeutic growth factors in these MSC NVs are still limited, which need further extension to meet the requirements of more effective wound healing.

Here, Fe‐MSC‐NVs were prepared and encapsulated in the inner hyaluronic acid (HA) core of the MN tips for accelerating angiogenesis. Because when the MSCs were treated with ferrum nanoparticles (Fe NPs), the capacity of MSC NVs in expressing therapeutic cytokines could be significantly enhanced. In addition, the PDA NPs were encapsulated in the outer methacrylated hyaluronic acid (HAMA) shell^[^ ^24^ ^]^ of the MN tips to overcome the adverse impacts from ROS‐derived oxidative stress, since PDA is proved to be an effective antioxidant agent based on its numerous reductive functional groups like catechol and imine.^[^ ^25^, ^26^, ^27^, ^28^ ^]^ It was demonstrated through in vitro and in vivo experiments that with the gradual degradation of HAMA patch tips in the skin, the PDA NPs could be sustainably released at the lesion to suppress the ROS‐induced inflammation reaction. At the same time, the Fe‐MSC‐NVs could significantly increase the human umbilical vein endothelial cells (HUVEC) migration, proliferation, and tube formation. Also, the combination of PDA NPs and Fe‐MSC‐NVs could further promote M2 macrophage polarization, thereby suppressing wound inflammation. The Fe‐MSC‐NVs/PDA MN patch exhibited excellent antiinflammatory, antioxidant, and pro‐angiogenic properties for accelerating diabetic wound closure. These results indicate that the proposed composite core‐shell MN patch is a promising candidate for wound healing.

## 2 Results and Discussion

### 2.1 Preparation and Characterization of Fe‐MSC‐NVs and PDA NPs

**Figure 2:**
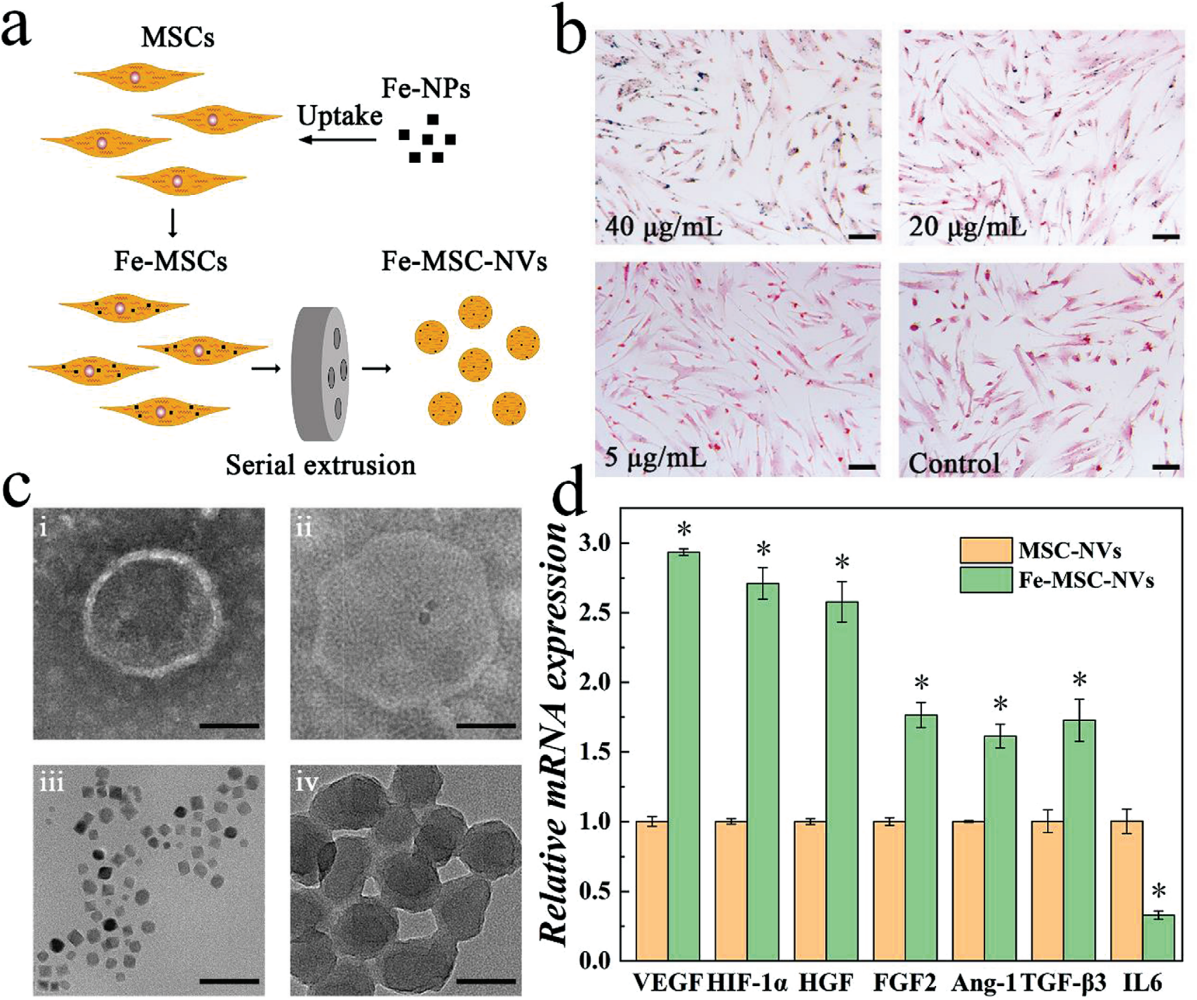
a) Schematic illustrations of preparation process of Fe‐MSC‐NVs. b) The Prussian blue staining of the MSCs when co‐cultured with 5, 20, and 40 µg mL^−1^ and free (Control) Fe NPs. The blue color: The Fe NPs in MSCs. Scale bars: 200 µm. c) TEM images of (i) MSC‐NVs, (ii) Fe‐MSC‐NVs, (iii) Fe NPs, and (iv) PDA NPs. Scale bars: 50 nm. d) Relative mRNA expression of VEGF, HIF‐1*α*, HGF, FGF2, Ang‐1, TGF‐*β*3, and IL‐6 in MSC‐NVs and Fe‐MSC‐NVs. n = 3, *p < 0.05 versus MSC‐NVs.

In a typical experiment, MSCs were co‐cultured with Fe NPs for 24 h, and the Fe‐MSCs were isolated through magnetic force (Figure S1a, Supporting Information). Fe‐MSC‐NVs were obtained by extruding the Fe‐MSCs through serial porous membranes to get functional MSC extracellular vesicles, as shown in **Figure** **2**a. Via the self‐polymerization approach of dopamine, PDA NPs were synthesized for the following experiments.^[^ ^25^ ^]^ The results of CCK‐8 assay and Calcein‐AM/PI staining showed that the 40 µg mL^−1^ Fe NPs and 80 µg mL^−1^ PDA NPs exhibited excellent biocompatibility and low toxicity (Figure S2a–d, Supporting Information). The results of Prussian blue staining suggested that the content of Fe NPs in MSCs augmented, when co‐culturing with the increasing concentration of Fe NPs (Figure 2b). Considering these results, 40 µg mL^−1^ Fe NPs were chosen in the following experiments. PDA NPs exhibited a high degree of uniformity, which were validated by the transmission electron microscope (TEM) (Figure 2c), with a diameter of 54.41±9.35 nm (Figure S1d, Supporting Information). Different from MSC‐NVs, Fe NPs could be seen in Fe‐MSC‐NVs. With more Fe NPs loading, the vesicles’ size gradually increased. The diameter and the polydispersity index (PDI) of Fe‐MSC‐NVs were 154.9±7.55 nm and 0.15±0.04, respectively, while the diameter and PDI of MSC‐NVs were 104.9±2.97 nm and 0.13±0.08 (Figure S1c, Supporting Information). Western blot also confirmed the exosomal markers (CD9 and CD63) in Fe‐MSC‐NVs, validating the fabrication of MSC‐derived exosomes (Figure S1b, Supporting Information).

Ferrum could enhance the secretion of multiple growth factors, such as vascular endothelial growth factor (VEGF), hepatocyte growth factor (HGF), angiopoietin‐1 (Ang‐1), and fibroblast growth factor‐2 (FGF2) in MSCs (Figure S3, Supporting Information). All these factors could contribute to the proliferation and migration of endothelial cells, and increase the re‐epithelialization and collagen deposition at wound sites. Compared with MSCs, MSC‐derived NVs would exhibit enhanced therapeutic efficiency in animal models and avoid the limitations of storage difficulty and low viability of MSCs in materials. Real‐time quantitative PCR (RT‐qPCR) analysis was performed to study the expression of cytokines in samples. The expression of therapeutic growth cytokines, such as VEGF, HGF, Ang‐1, FGF2, hypoxia‐inducible factor‐1 alpha (HIF‐1*α*), and transforming growth factor‐*β*3 (TGF‐*β*3), was significantly increased in Fe‐MSC‐NVs (Figure 2d), which indicated that Fe‐MSC‐NVs were more conducive to promoting angiogenesis than pure MSC‐NVs. Besides, the decreased expression of inflammatory factor interleukin‐6 (IL‐6) meant the internalization of Fe NPs in Fe‐MSC‐NVs might not induce pro‐inflammatory effects. These results suggested that Fe‐MSC‐NVs up‐regulated multifunctional therapeutic cytokines expression with low pro‐inflammatory properties.

### 2.2 Preparation and Characterization of Core‐Shell MN Patch

**Figure 3:**
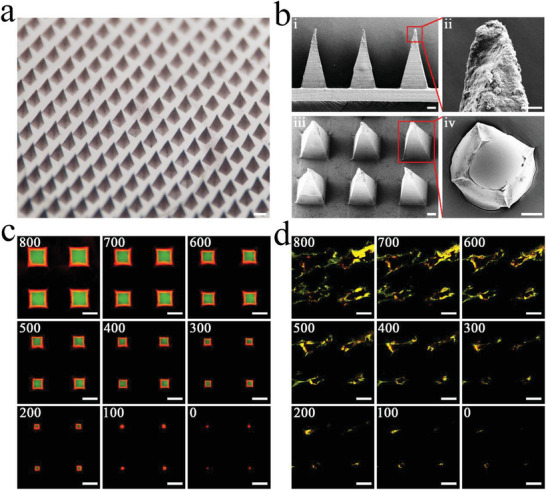
a) Optical image of Fe‐MSC‐NVs/PDA MN patch. Scale bar: 500 µm. b) SEM images of Fe‐MSC‐NVs/PDA MN patch; (i) Scale bars: 100 µm, (ii) Scale bars: 20 µm, (iii) Scale bars: 100 µm, (iv) Scale bars: 100 µm. c) Merge CLSM images of core‐shell MN at different depths: 0, 100, 200, 300, 400, 500, 600, 700, 800 µm. Scale bars: 200 µm. d) Ex vivo transdermal delivery of Fe‐MSC‐NVs in HA hydrogel and PDA NPs in HAMA hydrogel through the designed MN patch: Merge image at different depths (0, 100, 200, 300, 400, 500, 600, 700, and 800 µm) after the MN patch applied on the rat skin. Scale bars: 200 µm.

The multifunctional core‐shell MN patch, in which each MN exhibited a pyramid‐like shape with uniform dimension and sharp tip, was fabricated through the micromolding process (Figure S4, Supporting Information). Briefly, HAMA gel solution with PDA NPs was loaded into the microcavities of the polydimethylsiloxane (PDMS) mold, dried, and solidified through UV light irradiation for 1 min. Then, HA gel solution with Fe‐MSC‐NVs was filled into the cavities, and dried naturally overnight to get the soluble core. Pure HA gel solution was fulfilled to get a soluble backing layer. Finally, a complete core‐shell MN patch was obtained, with the PDA NPs shell and Fe‐MSC‐NVs core in tips. The MN patch contained a 20 × 20 MN array, and each MN possessed a quadrangular pyramid tip with a height of 860 µm, a base width of 360 µm, and a center‐center spacing of 700 µm (**Figure** **3**a). The microstructure of the MN patch was further observed by scanning electron microscopy (SEM) microscope (Figure 3b), which showed a complete morphology of MN array, with PDA NPs on the surface. The cross‐section SEM picture (Figure 3biv) confirmed an obvious core‐shell structure. To further study the core‐shell MN patch, DiO labeled Fe‐MSC‐NVs was dissolved in the inner HA core layer of tips, and red‐microspheres were dissolved in the outer HAMA shell layer of tips. The confocal scanning light microscopy (CSLM) pictures (Figure 3c and Figure S5a–c, Supporting Information) exhibited the red‐fluorescent shell and green‐fluorescent core filled in the MN patch, which confirmed the core‐shell MN patch was successfully fabricated. To evaluate the insertion ability of the core‐shell MN patch ex vivo, the above‐mentioned fluorescent patch was applied to the rats’ back skin. Figure 3d and Figure S5d,e, Supporting Information, showed that the core‐shell MN arrays could penetrate into the skin and deliver two fluorescent dyes into the skin simultaneously. In addition, these results demonstrated the MN arrays could deliver and enhance two separate contents absorption deeply in the skin at the same time.

### 2.3 Antioxidant Effect of PDA Nanoparticles

**Figure 4:**
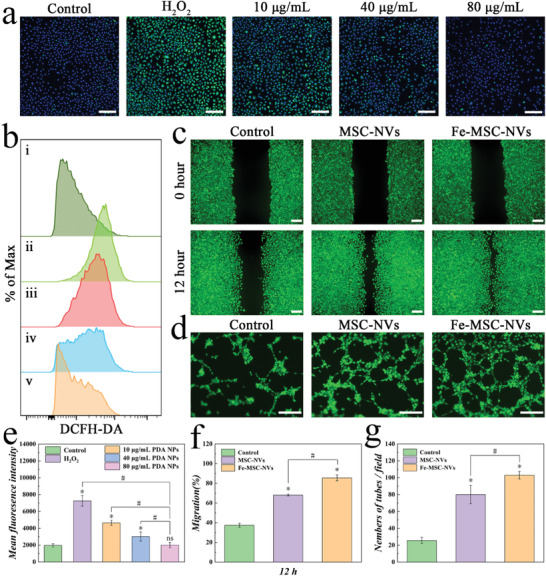
a) CLSM images of HUVEC cells using DCFH‐DA as the fluorescent probe: Control, H_2_O_2_, H_2_O_2_+10 µg mL^−1^ PDA NPs, H_2_O_2_+40 µg mL^−1^ PDA NPs, H_2_O_2_+80 µg mL^−1^ PDA NPs. Scale bars: 200 µm. b) The cellular uptake of DCFH‐DA by HUVEC with different concentrations of PDA NPs analyzed by flow cytometry: (i) Control; (ii) H_2_O_2_; (iii) H_2_O_2_+10 µg mL^−1^ PDA NPs; (iv) H_2_O_2_+40 µg mL^−1^ PDA NPs; (v) H_2_O_2_+80 µg mL^−1^ PDA NPs. c) Scratch assay images of HUVEC cultivated in the medium supplementary with PBS (Control), MSC‐NVs, or Fe‐MSC‐NVs. d) Matrigel angiogenesis assay in HUVEC cultured in the medium supplementary with PBS (Control), MSC‐NVs, or Fe‐MSC‐NVs. Scale bars: 200 µm. e) Corresponding quantitative analysis of ROS levels of HUVEC analyzed by flow cytometry. n = 5, ns p> 0.05. *p < 0.05 versus control. #p < 0.05. f) Cell migration quantification of HUVEC. n = 5, *p < 0.05 versus control. #*p* < 0.05. g) Matrigel angiogenesis assay in HUVEC. n = 5, *p < 0.05 versus control. #*p* < 0.05.

Oxidative stress is one of the reasons that prolongs the wound healing process, and excessive ROS may lead to endothelial cell dysfunction.^[^ ^29^ ^]^ Notably, ROS generated during the inflammatory process could be eliminated by PDA NPs, which acted as a radical scavenger owing to abundant phenol groups. To examine the ROS reduction effect of PDA NPs, HUVEC stimulated by H_2_O_2_ were selected as the model of oxidative stress injury, and the intracellular ROS was measured by a DCFH‐DA fluorescent probe. The flow cytometry results indicated the level of ROS gradually reduced with the increasing concentration of PDA NPs, indicating that PDA NPs treatment significantly reversed H_2_O_2_‐induced ROS formation in a dose‐dependent manner (**Figure** **4**b,e). Specifically, 80 µg mL^−1^ PDA NPs exhibited the strongest antioxidant activity and effectively suppressed the H_2_O_2_‐induced intracellular ROS production in HUVECs, which was also confirmed by CLSM (Figure 4a). The elimination of ROS to prevent skin tissue damage could be an important treatment of wound healing.

### 2.4 Promoting Angiogenesis by Fe‐MSC‐NVs

Newly formed vessels can deliver nutrition and oxygen to wounds so as to accelerate wound closure.^[^ ^30^ ^]^ Therefore, HUVEC migration, proliferation, and tube formation are essential steps during the angiogenesis process, determining the curative effect of diabetic wound healing. As shown in Figure 4c,f, Fe‐MSC‐NVs showed a higher migration rate (85.55±3.04%) than MSC‐NVs (68.14±1.05%) at an interval of 12 h. A higher proliferation rate (71.40±3.42%) was found in Fe‐MSC‐NVs group, versus 57.56±1.58% in MSC‐NVs group as shown in Figures S7 and S8, Supporting Information, determined by EdU assay. Meanwhile, Fe‐MSC‐NVs showed higher efficiency than MSC‐NVs in promoting HUVEC tube formation, characterized by more visible tube‐like structures and higher tube numbers, as shown in Figure 4d,g. The uptake of Fe‐MSC‐NVs in HUVEC cells was further confirmed by CSLM, which showed the DiD‐labeled Fe‐MSC‐NVs demonstrated a significant internalization in HUVEC cells (Figure S1e, Supporting Information). According to the previous research results, Fe‐MSC‐NVs could directly express more multifunctional cytokines known as the critical cytokines for angiogenesis, such as VEGF, FGF2, HGF, Ang‐1, HIF‐1*α*, TGF‐*β*3.^[^ ^31^ ^]^ HIF‐1*α* is essential in angiogenesis because it induces multiple angiogenic genes.^[^ ^32^, ^33^ ^]^ Besides, TGF‐*β*3 promotes collagen organization to reduce scars and improve healing.^[^ ^34^ ^]^ Therefore, these cytokines expressed in Fe‐MSC‐NVs collaboratively promote diabetic wound healing.

### 2.5 Polarization of Macrophages Toward M2 Phenotype

**Figure 5:**
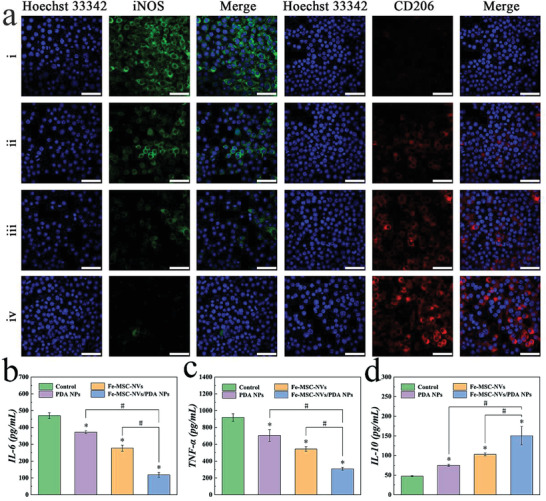
a) Immunofluorescence staining of the RAW 264.7 cells after being cocultured with (i) LPS, (ii) LPS/PDA NPs, (iii) LPS/Fe‐MSC‐NVs, or (iv) LPS/PDA NPs/Fe‐MSC‐NVs; iNOS (green): M1 macrophages; CD206 (red): M2 macrophages; Blue: nuclei. Scale bars: 50 µm. b–d) ELISA assay of (b) IL‐6, (c) TNF‐*α*, and (d) IL‐10 in cell culture supernatants. n = 5, *p < 0.05 versus control. #*p* < 0.05.

Macrophage polarization plays an important role in diabetic wound healing. M1 macrophages secret pro‐inflammatory mediators, while M2 macrophages secret anti‐inflammatory cytokines and therapeutic growth factors.^[^ ^35^ ^]^ M2 polarization could alleviate the inflammatory responses, thereby accelerating wound closure.^[^ ^36^ ^]^ As shown by the flow cytometry results in **Figure** **5** and Figure S9, Supporting Information, after LPS stimulation, RAW 246.7 cells transformed to M1 phenotype, with an increase in the percentage of iNOS‐positive and CD206‐negative cells in CD11b^+^F40/80^+^ cells. Either PDA NPs or Fe‐MSC‐NVs increased the percentage of iNOS‐negative and CD206‐positive cells in CD11b^+^F40/80^+^ cells (M2 phenotype), perhaps because Fe‐MSC‐NVs contained functional cytokines, and PDA NPs had plenty of biologically active functional groups such as C—OH, ‐N—H_2_, C═O, which involved in the conjugation of various biomolecules to contribute to its biological activity. In addition, the combination of PDA NPs and Fe‐MSC‐NVs showed the highest expression of the M2 phenotype (40.08±2.17%) and the lowest expression of the M1 phenotype (6.43±0.91%), which was more conducive to the repair of diabetic wounds. Furthermore, the polarization of RAW 246.7 cells was also confirmed by immunofluorescence staining (Figure 5a), which was in accord with the flow cytometry results. The ELISA analysis of the cell culture supernatant indicated that the combination of PDA NPs and Fe‐MSC‐NVs showed a significant increase of anti‐inflammatory factor IL‐10 (150.44±23.42 pg mL^−1^) than the control group (47.34±1.45 pg mL^−1^) (Figure 5d). IL‐10 was one of the most important anti‐inflammatory factors secreted by M2 macrophages. In contrast, the pro‐inflammatory mediators (IL‐6 and TNF‐*α*) secreted by M1 macrophages were inhibited efficiently, which showed 118.27±14.10 pg mL^−1^ versus 470.32±17.47 pg mL^−1^ and 308.56±17.21 pg mL^−1^ versus 918.92±44.35 pg mL^−1^ in the Fe‐MSC‐NVs/PDA NPs group and control group (Figure 5b,c). Thus, it was verified that the combination of PDA NPs and Fe‐MSC‐NVs inhibited M1 phenotype and promoted the polarization of macrophages toward M2 phenotype.

### 2.6 The Release Study of MN Patch

Persistent hyperglycemia and infection can lead to the excessive accumulation of ROS during diabetic wound healing, impairing wound healing by causing cell death and systemic injury. The sustained antioxidant effect of removing ROS is critical for diabetic wounds.^[^ ^29^ ^]^ In this research, Fe‐MSC‐NVs/PDA MN was fabricated for diabetic wound healing. The results of CCK‐8 assay and Calcein‐AM/PI staining showed Fe‐MSC‐NVs/PDA MN and its contents exhibited good biocompatibility and low toxicity (Figure S2e,f, Supporting Information). HA has good degradation characteristics and HAMA has been proved to degrade in the presence of hyaluronidase.^[^ ^37^ ^]^ HAMA/HA MN patch or only HA MN patch was placed in the PBS containing 100 units of hyaluronidase at 37 °C. Figure S6, Supporting Information, shows only HA patch would degrade in 2 h, and HAMA layer seemed to degrade more slowly. After 72 h, most of the HAMA layer changed to fragments. By studying the release of two fluorescent dyes from the core‐shell patch, it was found the MN patch was assembled by a rapid‐released core layer and a slow‐released shell layer. The in vitro release experiments (Figure S5f, Supporting Information) showed that the soluble HA layer could release the content quickly. The cumulative release curve could reach 81.72±5.20% release within 1 h. Besides, the HAMA layer showed a sustained release rate. The cumulative release reached 82.06±5.07% after 72 h. This design was conducted to sustain the release of PDA NPs from the shell layer to suppress ROS during all stages of wound healing. Meanwhile, according to the excellent adhesion effect of PDA NPs, storing PDA NPs in the shell layer could improve the adhesion of the MN patch to the wounds (Figure S10b,c, Supporting Information). The soluble core layer containing Fe‐MSC‐NVs was beneficial to release Fe‐MSC‐NVs quickly and entirely for more efficient anti‐inflammatory and angiogenic effects. It could be found that Fe‐MSC‐NVs/PDA MN was multifunctional and practical in multiple stages of diabetic wound healing.

### 2.7 In Vivo Diabetic Wound Healing

**Figure 6:**
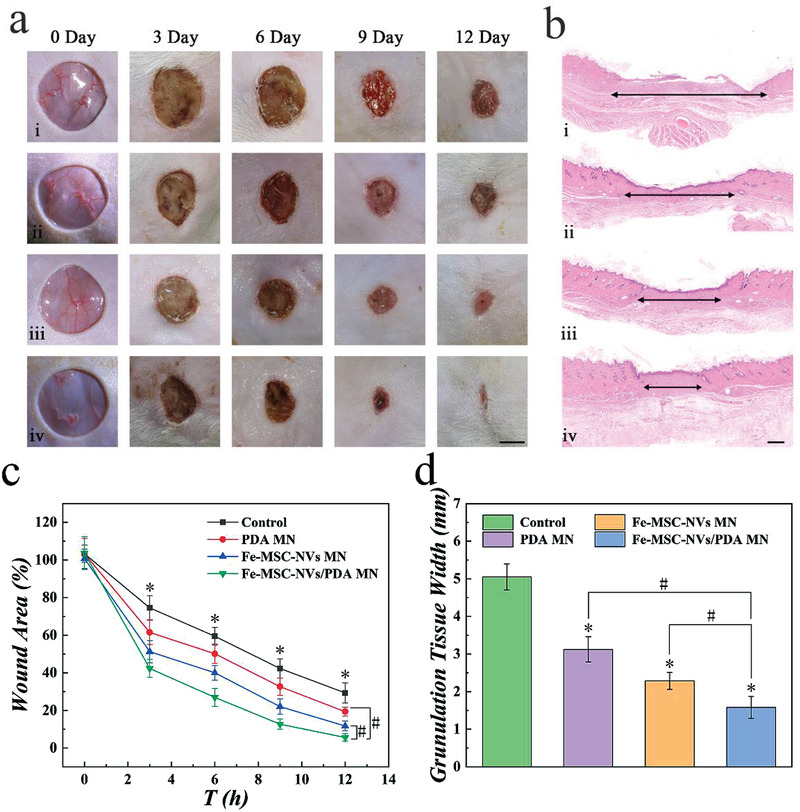
The efficiency of the core‐shell MN patch on wound healing. a) The photos of wound closure in different treatment groups: control group, PDA MN, Fe‐MSC‐NVs MN, and Fe‐MSC‐NVs/PDA MN on days 0, 3, 6, 9, and 12. Scale bar: 5 mm. b) HE staining of the wounds in different treatment groups: control group, PDA MN, Fe‐MSC‐NVs MN, and Fe‐MSC‐NVs/PDA MN on day 12. Scale bars: 0.5 mm. c) Average wound area in different treatment groups during the wound healing processes from day 0 to day 12, n = 6, *p < 0.05 versus treatment group. #*p* < 0.05. d) Corresponding quantitative analysis of granulation tissue width in different treatment groups on day 12, n = 6, *p < 0.05 versus control. #*p* < 0.05.

Fe‐MSC‐NVs/PDA MN patch, PDA MN patch, Fe‐MSC‐NVs MN patch, and PBS as the control group were performed to evaluate their curative effect in wound healing in vivo. Animals were divided into four groups randomly, and the wound model on the back of diabetic rats was performed at a diameter of 1.5 cm. The wound healing processes in these groups were recorded on days 0, 3, 6, 9, and 12. The patches applied on the wounds are shown in Figure S10, Supporting Information. It was found that with the gradual degradation of the patch, the appearance of the wounds was not affected. The effect observation was performed by calculating the area of wound closure rate with the Image‐J software. The results demonstrated that the Fe‐MSC‐NVs/PDA MN patch was more effective in promoting wound healing than other groups (**Figure** **6**a,b). The wounds were almost healed by Fe‐MSC‐NVs/PDA MN patch, with only 5.62±2.03% remaining wound area after treatment on day 12. However, 29.35±5.36% wound area still existed in the control group. In contrast, the PDA MN patch group and Fe‐MSC‐NVs MN patch group demonstrated 19.37±2.37% and 11.76±2.66% wound area remained, respectively (Figure 6c).

**Figure 7:**
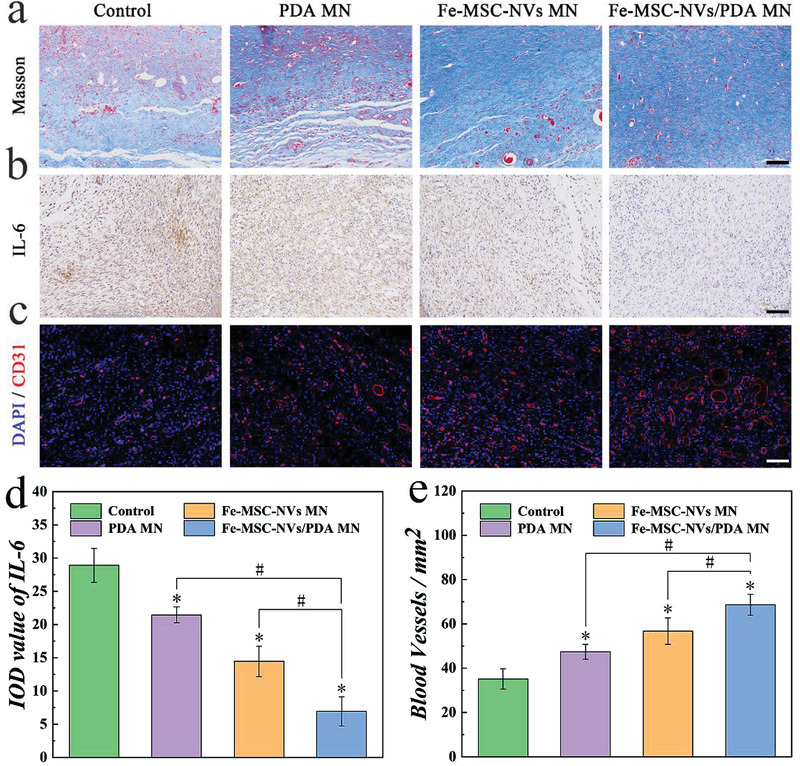
The efficiency of the core‐shell MN patch on wound healing in diabetic rats on day 12. a) Masson staining for collagen deposition. Scale bar: 100 µm. b) Immunohistochemical staining of IL‐6. Scale bar: 100 µm. c) Immunofluorescence staining of CD31 of granulation tissues in different groups. Scale bar: 200 µm. d) Corresponding quantitative analysis of the mean value of the IOD for immunohistochemical staining of IL‐6 in different treatment groups. n = 6, *p < 0.05 versus control. #*p* < 0.05. e) Corresponding quantitative analysis of CD31 microvessel density in different treatment groups. n = 6,*p < 0.05 versus control. #*p* < 0.05.

Hematoxylin‐eosin (HE) staining and Masson staining were performed for the investigation of histological changes of the wounds (Figure S12, Supporting Information). Collagen was dramatically reduced in the damaged skin tissue, leading to poor wound healing and impaired tissue remodeling. On the 12th day, only a few collagen fibers appeared in the control group, which were loose and disordered. The Fe‐MSC‐NVs/PDA MN patch group showed more collagen deposition, with the denser, thicker, and better‐arranged collagen fibers in skin tissues, as shown in **Figure** **7**a. Immunohistochemical staining of IL‐6 showed that the application of Fe‐MSC‐NVs/PDA MN patch alleviated the inflammatory state significantly (Figure 7b,d). HE staining further indicated that Fe‐MSC‐NVs/PDA MN patch group had a shorter granulation tissue width than other groups (Figure 6d), which was consistent with the previous results of wound closure rate. The HE staining on days 3, 6, and 9 further demonstrated that the Fe‐MSC‐NVs/PDA MN patch group displayed the best curative effect (Figure S12a, Supporting Information). The wound crust (scab) appeared to cover the wound in the early stage of wound healing, especially in the control group (Figure S13a,c, Supporting Information); while the complete epidermal layer was not formed until day 9 in the Fe‐MSC‐NVs/PDA MN patch group (Figure S13b,c, Supporting Information). The control group could not form a complete epithelial tissue on day 12. The treating groups had formed the re‐epithelialization process, and the Fe‐MSC‐NVs/PDA MN patch group showed the largest epithelial thickness (Figure S11, Supporting Information).

The polarization of macrophages affects the inflammation stage and granulation stage of wound healing (approximately 3–10 days).^[^ ^38^ ^]^ And M2 macrophages inhibit inflammation and promote tissue regeneration. RT‐qPCR was performed to measure the M1 macrophage marker of iNOS and the M2 macrophage marker of arginase‐1 (Arg‐1) at the wound site to assess macrophage polarization (Figure S18, Supporting Information). The Fe‐MSC‐NVs/PDA MN patch had the best inhibitory effect on M1 macrophages on the 3rd and 6th days. There was no significant difference between Fe‐MSC‐NVs/PDA MN patch group and the Fe‐MSC‐NVs MN patch group, which demonstrated that these two groups could effectively inhibit inflammation on the 9th day. And the Fe‐MSC‐NVs/PDA MN patch group showed better inhibition than the PDA MN patch group and control group. Although either PDA NPs or Fe‐MSC‐NVs had anti‐inflammatory effects, the combination of PDA NPs and Fe‐MSC‐NVs in the MN patch maximized therapeutic effectiveness. By suppressing M1 macrophages, the expression of M2 macrophages increased gradually on days 3, 6, and 9. The Fe‐MSC‐NVs/PDA MN patch group showed the best M2 polarization these days compared with other groups. The immunohistochemical staining of IL‐6, TNF‐*α*, and IL‐10 also showed similar results (Figures S14–S17, Supporting Information).

Angiogenesis is an indispensable step in the remodeling phase of wound healing. M2 macrophages could secrete VEGF and increase the proliferation of vascular endothelial cells to promote angiogenesis at the wound site.^[^ ^39^ ^]^ Fe‐MSC‐NVs also promoted angiogenesis by increasing the proliferation, migration, and tube formation of HUVEC. CD31 plays a key role in angiogenesis, which is a typical marker of vascular endothelial cells. The expression of CD31 was highest in the Fe‐MSC‐NVPDA MN patch group on days 3, 6, 9, and 12 (Figure 7c,e and Figures S19 and S20, Supporting Information). This result indicated that the Fe‐MSC‐NVs/PDA MN patch showed better pro‐angiogenic capability. In summary, the Fe‐MSC‐NVs/PDA MN patch had good antiinflammation activity, antioxidant capacity, and angiogenic properties, demonstrating great potential in accelerating diabetic wound closure.

## 3 Conclusion

In conclusion, we have fabricated a novel degradable and biocompatible core‐shell MN patch with Fe‐MSC‐NVs and PDA NPs encapsulated in the core and shell of tips, respectively, for promoting wound healing. The characteristic core‐shell structure gave the MN patch room for further multi‐functionalization, since hierarchical structures were capable of loading different functional components in distinct compartments. The core‐shell MN patch had several characteristics: First, owing to the existence of Fe‐MSC‐NVs in the core area, the MN patch could effectively accelerate angiogenesis at the wound site. Fe‐MSC‐NVs contained multiple growth factors, such as VEGF, HIF‐1*α*, HGF, FGF2, etc. All these factors would contribute to the proliferation and migration of endothelial cells, and increase the re‐epithelialization and collagen deposition at wound sites. Compared with traditional MSC‐exosomes, these artificially modified MSC‐NVs can be more functional, loading many kinds of cargos, including genetic substances, proteins, or small molecules to show a greater curative effect. Second, through encapsulating PDA NPs in the shell layer, the MN patch could overcome the adverse impacts from ROS‐derived oxidative stress, including the hindrance of cell proliferation, spread, and transplantation. Third, the combination of Fe‐MSC‐NVs and PDA NPs could induce macrophage polarization changes from M1 to M2 phenotype, accompanied by the increased secretion of anti‐inflammatory cytokines and inhibition of pro‐inflammatory mediators. Last, PDA exhibited similar properties to the mussel secreted adhesive protein, which could adhere to any substrate to achieve long‐lasting adhesion.^[^ ^40^ ^]^ The MN patch was specially designed to load the PDA NPs in the outer layer of the MN, which could increase the adhesion to the tissues and make the application more reasonable and convenient. Through in vivo tests, it was confirmed that the core‐shell Fe‐MSC‐NVs/PDA MN patch had a remarkable curative effect in promoting collagen regeneration, anti‐oxidation, anti‐inflammation, and pro‐angiogenesis in the diabetic wound healing models. Therefore, the Fe‐MSC‐NVs/PDA MN patch is expected to become a promising candidate for accelerating wound healing, showing great application potentials in various related biomedical fields.

## 4 Experimental Section

### Materials

HA (10 kDa) was purchased from Dalian Meilun Biotechnology Co., Ltd. (China). 150 kDa Methacrylated hyaluronic acid (HAMA) was purchased from EngineeringForLife company (Suzhou, China). 2‐hydroxy‐2‐methylpropiophenone (HMPP) and dopamine hydrochloride were purchased from Sigma‐Aldrich (Shanghai, China). Fe NPs were obtained from Nanjing Nanoeast Biotech. Co. Ltd. (China). Red‐microspheres (≈50 nm) were purchased from the Invitrogen Company (Thermo Fisher Scientific). DCFH‐DA (2“,7”‐dichlorodihydrofluorescein diacetate) was purchased from MedChemExpress company (NJ, USA). Calcein AM, PI, Hoechst 33 342, BeyoClick EdU Cell Proliferation Kit, and Cell Counting Kit‐8 (CCK‐8) were purchased from Beyotime (China). DiD Perchlorate (The 1,1’‐dioctadecyl‐3,3,3’,3’‐tetramethylindodicarbocyanine,4‐chlorobenzenesulfonate salt) and DiO perchlorate (3,3‐dioctadecyloxacarbocyanine perchlorate) were purchased from Yeasen Biotechnology Co., Ltd. (Shanghai, China). Mouse ELISA kits were purchased from BioLegend Company (San Diego, CA, USA). DMEM/F12 and DMEM (high glucose) were purchased from Wisent Inc. (Canada). All reagents were of analytical grade.

### Cell Culture and Animals

MSCs isolated from fresh human umbilical cords were prepared through previous methods.^[^ ^41^ ^]^ The Cell culture medium of MSCs was DMEM/F12. HUVEC and RAW 246.7 were cultured in DMEM (high glucose). All the cells were used in the logarithmic growth phase, cultured with 10% fetal bovine serum and 1% penicillin‐streptomycin double antibiotics at 37  °C with 5% CO_2_.

Male Sprague−Dawley rats (SD) weighing 180–220 g for in vivo experiment were purchased from Nanjing Medical University (Nanjing, China) and maintained in the Animal Laboratory Center of the Affiliated Drum Tower Hospital of Nanjing University Medical School. The experimental protocol was approved by the Animal Care and Use Committee of the Affiliated Drum Tower Hospital of Nanjing University Medical School.

### Synthesis of PDA NPs

PDA NPs were synthesized via a classical Stöber method. In detail, after the mixture (concentrated NH_4_OH, ethanol, and water) was stirred for 30 min, dopamine hydrochloride aqueous solution was added at room temperature. After 1 day under stirring, PDA NPs were obtained, washed by distilled water, and freeze‐dried overnight. CCK‐8 assay and Calcein‐AM/PI staining were used to determine the cytotoxicity of the PDA NPs. TEM (JEOL JEM‐2100 microscope) technique was performed to visualize the shape and size of PDA NPs. The particle size distribution of PDA NPs was statistically analyzed through TEM images using Image‐J software.

### Characterization of Fe‐MSCs

TEM technique was used to visualize the shape and size of the Fe NPs. Different concentrations of the Fe NPs of 0, 10, 20, 40, and 80 µg mL^−1^ were treated with MSCs in the culture medium to evaluate the cytotoxicity. CCK‐8 assay was used to access the viabilities of the Fe NPs‐treated MSCs. Calcein‐AM and PI staining were used as a viability assay to validate the cytotoxicity of the Fe NPs in MSCs. Prussian blue staining was performed to observe the internalized Fe NPs in Fe‐MSCs. The MSCs were co‐cultured with Fe NPs for 24 h. Then TRIzol reagent was used to isolate the total RNA of Fe‐MSCs or MSCs. RNA quality and expression were evaluated using a StepOne RT‐PCR System (Applied Biosystems), following data analyzed by the comparative Ct (ΔΔCt) method, using GAPDH as the reference.

### Preparation and Characterization of Fe‐MSC‐NVs

After MSCs co‐cultured with Fe NPs for a whole day, Fe‐MSCs were collected by magnetic force. Collected Fe‐MSCs were resuspended in PBS. An Avanti mini‐extruder was used to extrude Fe‐MSCs through 10, 5, 1, and 0.4 µm Whatman membrane filters to obtain Fe‐MSC‐NVs. MSC‐NVs were obtained by the same method. MSC‐NVs and Fe‐MSC‐NVs were characterized by TEM. The size distributions of MSC‐NVs and Fe‐MSC‐NVs were analyzed by a Malvern Zetasizer NanoZS. The total protein concentration of MSC‐NVs and Fe‐MSC‐NVs was analyzed through Pierce BCA Protein Assay Kit (Thermo). The mRNA expression of MSC‐NVs and Fe‐MSC‐NVs was evaluated through RT‐qPCR. Western blot method was applied to analyze the exosomal markers CD9 (Proteintech) and CD63 (Proteintech) in MSC‐NVs and Fe‐MSC‐NVs. The DiD labeled Fe‐MSC‐NVs were used to evaluate the internalization of Fe‐MSC‐NVs in cells by CLSM.

### Fabrication of MN Patch

HAMA hydrogel (0.1 g mL^−1^), HMPP (1%, v/v), and PDA NPs (80 µg) were mixed in aqueous solution to make the outer layer of the tips. After drying overnight and solidifying the tips through UV irradiation for 60 s, HA (0.3 g mL^−1^) and Fe‐MSC‐NVs (50 µg) were entirely filled into the quadrangular pyramid microcavities of the mold to prepare the tip solution and dried for 12 h. The backing layer solution (0.3 g mL^−1^ HA hydrogel) was then filled and dried for 12 h. The resultant MN patch was obtained by demolding. The optical images of MN array were obtained through a Canon 5D Mark II digital camera. SEM photos were taken by a Zeiss Ultra Plus SEM. The CLSM images were taken by an Olympus FV3000 confocal microscope using fluorescent dyes as a demonstration, with red‐microspheres (≈50 nm) in the outer HAMA layer and DiO‐labeled Fe‐MSC‐NVs loaded in the inner HA layer.

### Ex Vivo Skin Insertion Test

Red‐microspheres and DiO‐labeled Fe‐MSC‐NVs loaded core‐shell MN patches were inserted into the living rat skins for 5 min to observe skin penetration after insertion. MN insertion sites were observed using an Olympus FV3000 confocal microscope to examine the skin penetration capability of the MN patch. 3D CLSM images were remodeled by continuous z‐stack confocal images.

### In Vitro Cell Experiments

The biocompatibility of biomaterials was evaluated by CCK‐8 assay and Calcein‐AM/PI staining. Each experiment with five replicates was performed for CCK‐8 assay, cell migration, cell proliferation, tube formation, antioxidant efficiency of polydopamine, and M2 polarization of macrophages.

### Cell Migration Experiment

2 × 10^5^ HUVEC cells/well were plated into a 6‐well plate to reach confluence. HUVEC monolayer was scratched by a tip, then washed with PBS three times. The HUVEC cells were treated with PBS, MSC‐NVs (50 µg/well), or Fe‐MSC‐NVs (50 µg/well). The fluorescence images of the HUVEC cells labeled by Calcein AM were taken at 0 and 12 h using an Olympus inverted microscope. The cell migration percentage was calculated as the following formula: migration area (%) = [(*A*_1_ − *A*_2_)/*A*_1_] × 100%, where *A*_1_ denotes the initial area, *A*_2_ denotes the remaining area after culture for 12 h.

### Proliferation Assay

Cell proliferation experiment was performed using a BeyoClick EdU Cell Proliferation Kit, based on the 5‐ethynyl‐2’‐deoxyuridine (EdU) as the synthetic analogs on the DNA synthetic processes. The CLSM images were visualized by an Olympus FV3000 confocal microscope. EdU‐positive cells were calculated as the following formula: EdU‐positive cells % = (EdU‐positive cells, red)/(Hoechst 33342‐positive cells, blue)  ×  100%.

### Tube Formation Assay

2 × 10^4^/well HUVEC were incubated on the polymerized Matrigel treated with PBS, MSC‐NVs (50 µg/well), or Fe‐MSC‐NVs (50 µg/well). After 6 h of harvest, the HUVEC cells were labeled by Calcein AM. The fluorescent photos were taken by a Leica inverted fluorescence microscope. The total tubes or nodes were measured by Image‐J software.

### Antioxidant Efficiency of Polydopamine

The cell‐permeable DCFH‐DA was applied to measure the ROS levels in cells. HUVEC were first cultured in a 6‐well plate with the various treatments of PDA NPs (0, 10, 40, and 80 µg mL^−1^) for a whole day, and then cultured with 500 µm H_2_O_2_ for 4 h. After PBS washing three times, HUVEC were treated with DCFH‐DA in the medium for 0.5 h in the cell incubator. The CLSM images were visualized by an Olympus FV3000 confocal microscope. The cells were analyzed on a BD LSRI Fortessa flow cytometer analyzer to quantify the results, with FlowJo X Software to deal with the data.

### M2 Polarization of Macrophages

RAW 264.7 macrophages cultured in 12‐well plates were classified into four groups: 1) LPS group (control group): Cells cultured with 200 ng mL^−1^ LPS; 2) PDA NPs: Cells cultured with 80 µg mL^−1^ PDA NPs, and 200 ng mL^−1^ LPS; 3) Fe‐MSC‐NVs: Cells cultured with 50 µg mL^−1^ Fe‐MSC‐NVs and 200 ng mL^−1^ LPS; 4): PDA NPs/Fe‐MSC‐NVs: Cells cultured with 50 µg mL^−1^ Fe‐MSC‐NVs, 80 µg mL^−1^ PDA NPs, and 200 ng mL^−1^ LPS. Each group was cultured for 24 h. RAW 264.7 cells were collected and stained with PE‐conjugated CD206 (Invitrogen), APC conjugated iNOS (Invitrogen), FITC conjugated F40/80 (Invitrogen), percp‐cy5.5 conjugated CD11b (Biolegend), and fixable viability dye‐eFluor 506 (Invitrogen). The cells were analyzed by flow cytometry. The IL‐6, TNF‐*α*, and IL‐10 levels in the cell culture supernatants were analyzed using ELISA kits. RAW 264.7 cells stained with anti‐CD206 antibodies (Invitrogen) or anti‐iNOS antibodies (Invitrogen) were visualized by an Olympus FV3000 confocal microscope.

### The Release Study of MN Patch

The MN stimulated release experiment was carried out using DiO‐labeled Fe‐MSC‐NVs in the inner layer of tips and red‐microspheres in the outer layer of tips as the demo. The MN was kept in PBS buffer and equilibrated at 37 °C to evaluate the release profile. The fluorescent intensity was detected by a microplate spectrofluorometric reader (Tecan Spark, Switzerland). To access the degradation properties of MN patch, the HAMA/HA core‐shell MN patch or the only HA MN patch was placed in the PBS containing 100 units of hyaluronidase (Sigma) at 37 °C. The solution was changed with fresh hyaluronidase‐contained buffer solution every 48 h. The photographs of MN patches at different times were taken by an Olympus microscope.

### In Vivo Wound Healing

Type I diabetes mellitus was conducted by intraperitoneally injecting streptozotocin (STZ) citrate buffer solution into mate SD rats (65 mg kg^−1^). After about 14 days, STZ‐treated rats with blood sugar higher than 16.7 mmol L^−1^ were anesthetized by isoflurane. After shaving and cleansing the back, a full‐thickness rounded skin wound with a diameter of 1.5 cm was prepared on the back. Animals were divided into four equal groups stochastically. Each group was treated by different interventions of PBS, PDA MN, Fe‐MSC‐NVs MN, and Fe‐MSC‐NVs/DA MN, respectively. The patches were applied every three days since the material of the MN would degrade. The images of wound closure were taken on 0, 3, 6, 9, and 12 days. Every three days, the rats were sacrificed. The full‐thickness skin tissues were collected and stored in paraformaldehyde fix solution for Hematoxylin and eosin (HE) staining, Masson's trichrome staining, immunohistochemical staining with IL‐6 antibody (Abcam), TNF‐*α* antibody (Santa Cruz), IL‐10 antibody (Proteintech), as well as immunofluorescence staining, which was conducted with CD31 antibody (Abcam) overnight at 4 °C to evaluate inflammatory state and neovascularization. Image Pro Plus 6.0 software was used to calculate the mean value of the integrated optical density (IOD) for IHC staining of IL‐6, TNF‐*α*, IL‐10. CD31 intensity was quantified using Image‐J software to determine blood vessel density. The mRNA expression of iNOS and Arg‐1 on the wounds on days 3, 6, 9 were evaluated through RT‐qPCR, and quantified data were normalized to the rat skin on day 0.

### Statistical Analysis

Statistical analysis was performed with the aid of GraphPad Prism 8 software. All the data were presented as mean ± SD. Unpaired t‐tests were used to compare statistically significant differences between two groups, while one‐way analysis of variance (ANOVA) was utilized to compare multiple groups. A *P* value of less than 0.05 was considered statistically significant.

## Conflict of Interest

The authors declare no conflict of interest.

## Author Contributions

W.J.M. and X.X.Z. contributed equally to this work. Y.J.Z. and L.Y.S. conceived the idea and designed the experiment; W.J.M. and R.F. conducted experiments and data analysis; J.J.G. and L.W.L. assisted with cell culture; W.J.M. and Y.X.L. wrote the manuscript; X.X.Z. revised the manuscript.

## Supporting information

Supporting Information

## Data Availability

Data sharing is not applicable to this article as no new data were created or analyzed in this study.
