# Supplementary material for: Polydopamine Decorated Microneedles with Fe‐MSC‐Derived Nanovesicles Encapsulation for Wound Healing
Source: Adv Sci (Weinh). 2022 Mar 10;9(13):2103317. doi: 10.1002/advs.202103317 (PMC9069192; doi:10.1002/advs.202103317)
Supplement: Supplementary file 1 — Supporting Information [file ADVS-9-2103317-s001.pdf]

ADVANCED SCIENCE
Open Access

Supporting Information

for *Adv. Sci.*, DOI 10.1002/advs.202103317

Polydopamine Decorated Microneedles with Fe-MSC-Derived Nanovesicles Encapsulation for Wound Healing

*Wenjuan Ma, Xiaoxuan Zhang, Yuxiao Liu, Lu Fan, Jingjing Gan, Weilin Liu, Yuanjin Zhao and Lingyun Sun**

Supporting Information

Polydopamine decorated microneedles with Fe-MSC-derived nanovesicles encapsulation for wound healing

Wenjuan Ma, Xiaoxuan Zhang, Yuxiao Liu, Lu Fan, Jingjing Gan, Weilin Liu,

Yuanjin Zhao and Lingyun Sun*

Dr. W. Ma, Prof. Y. Zhao, Prof. L. Sun

Department of Rheumatology and Immunology, China Pharmaceutical University

Nanjing Drum Tower Hospital, Nanjing 210008, China

Dr. W. Ma, Dr. J. Gan, Dr. W. Liu, Prof. Y. Zhao, Prof. L. Sun

Department of Rheumatology and Immunology, The Affiliated Drum Tower Hospital of Nanjing University Medical School, Nanjing 210008, China;

Email: lingyunsun@nju.edu.cn;

Dr. X. Zhang, Y. Liu, Dr. L. Fan, Prof. Y. Zhao

State Key Laboratory of Bioelectronics, School of Biological Science and Medical Engineering, Southeast University, Nanjing 210096, China

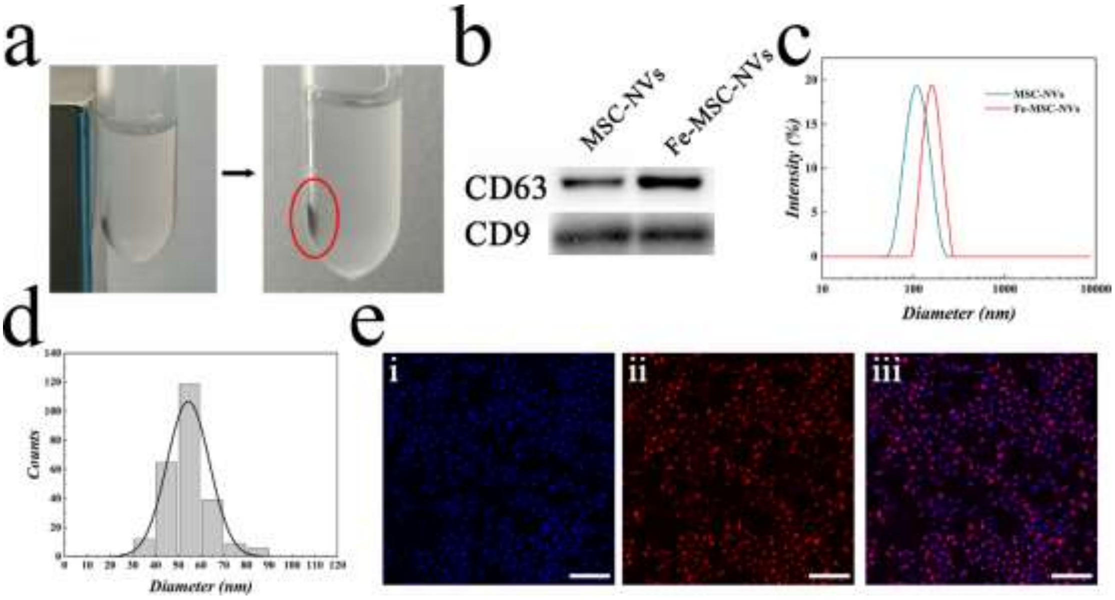

Figure S 1 (a) Magnetic attraction of Fe-MSCs. (b) The exosomal markers (CD9 and CD63) in MSC-NVs and Fe-MSC-NVs were analyzed by Western blot. (c) The particle size of MSC-NVs and Fe-MSC-NVs. (d) Corresponding size distribution histogram of PDA NPs derived from TEM images. (e) Evaluation of cellular uptake of DiD labeled Fe-MSC-NVs in HUVEC: (i) DAPI, (ii) DiD labeled Fe-MSC-NVs, (iii) Merge. Scale bars: 200 μm.

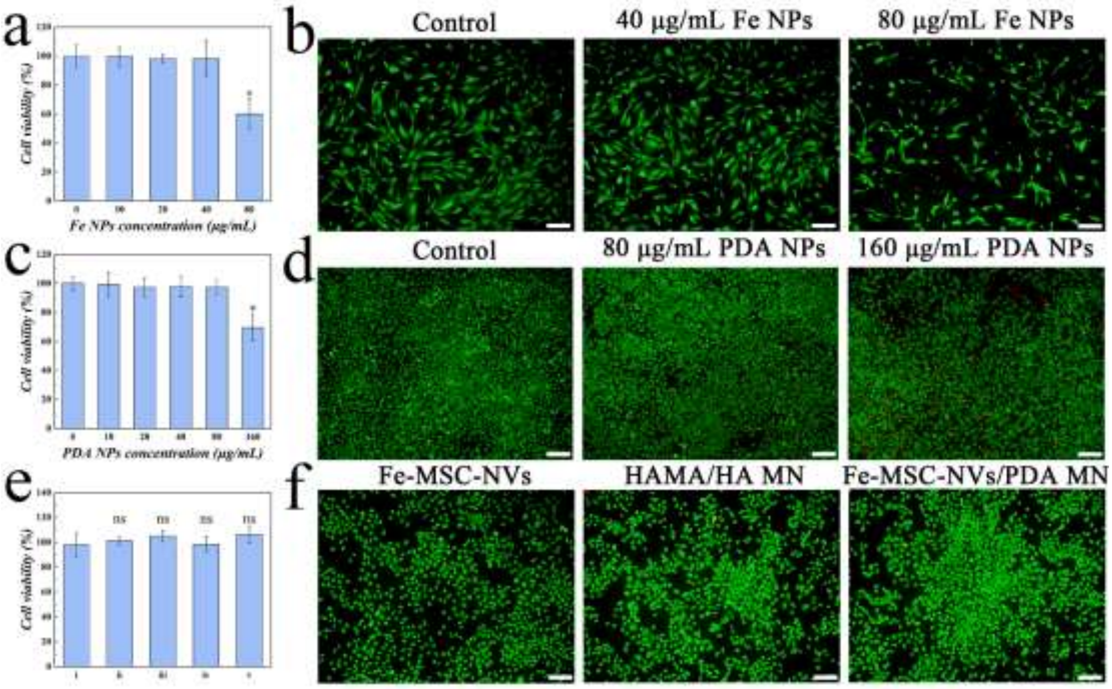

Figure S 2 (a) The viabilities of MSCs incubated with various amounts of Fe NPs for 24 h. n = 5,* $p < 0.05$ versus control. (b) Calcein AM/PI staining of MSCs in PBS (Control), 40, 80 μg/mL Fe NPs. (c) The viabilities of HUVEC incubated with various amount of PDA NPs for 24 h. n = 5, * $p < 0.05$ versus control. (d) Calcein AM/PI staining of HUVEC in PBS (Control), 80, 160 μg/mL PDA NPs. (e) The viabilities of HUVEC incubated for 24 h with different groups of (i) PBS (Control), (ii) 50 μg/mL MSC-NVs, (iii) 50 μg/mL Fe-MSC-NVs, (iv) HAMA/HA MN, (v) Fe-MSC-NVs/PDA MN. n = 5, ns $p > 0.05$. (f) Calcein AM/PI staining of HUVEC in 50 μg/mL Fe-MSC-NVs, HAMA/HA MN, Fe-MSC-NVs/PDA MN. Scale bars: 100 μm.

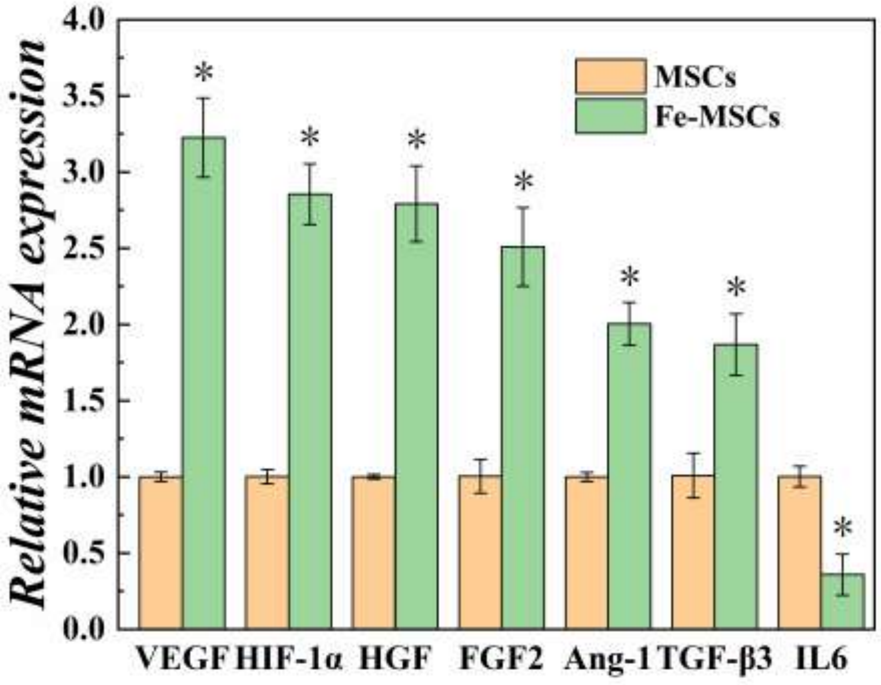

Figure S 3 Relative mRNA expression of VEGF, HIF-1α, HGF, FGF2, Ang-1, TGF-β3, and IL-6 in MSCs and Fe-MSCs. n = 3, * $p < 0.05$ versus MSCs.

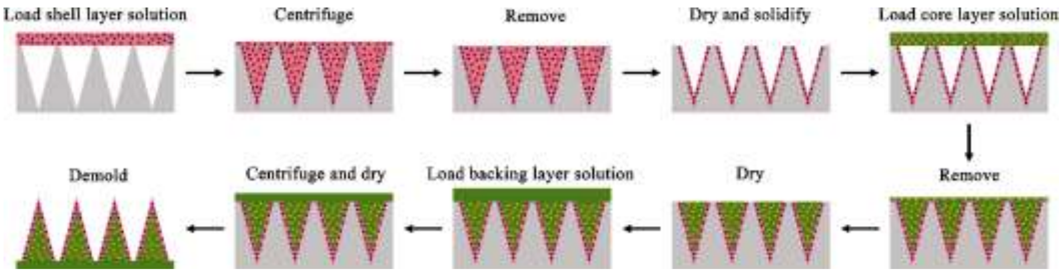

Figure S 4 Schematic illustration of the fabrication of the core-shell MNs.

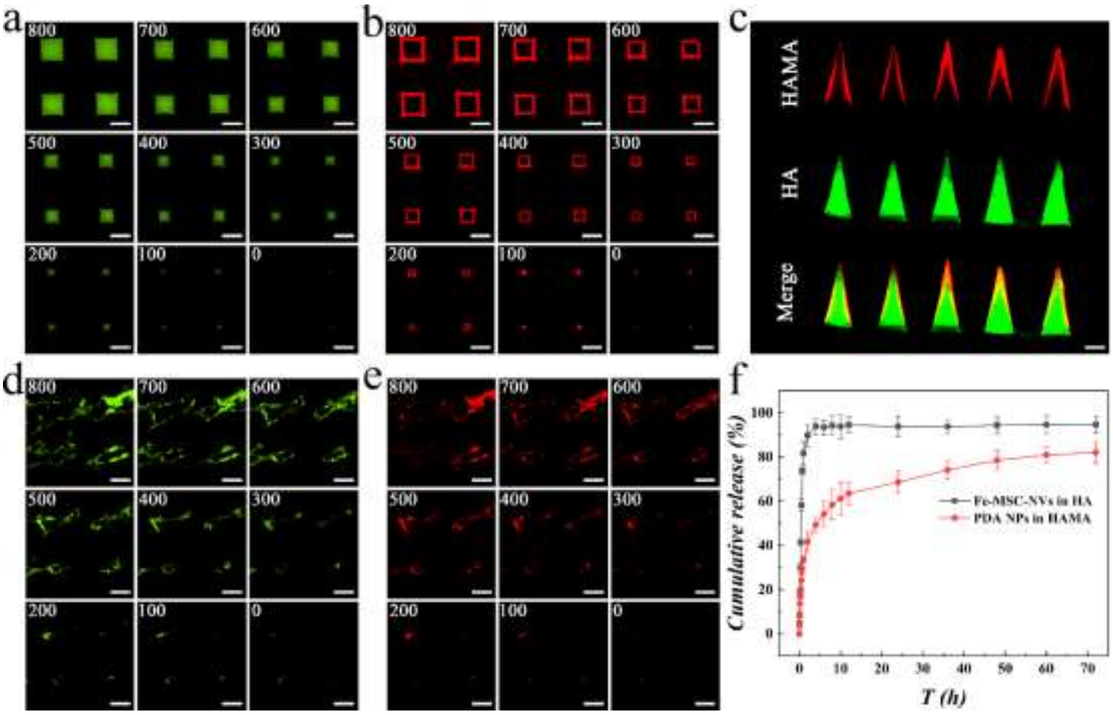

Figure S 5 CLSM images of (a) DiO-labled Fe-MSC-NVs layer and (b) red-microspheres layer at varying depths: 0, 100, 200, 300, 400, 500, 600, 700, 800 μm. Scale bars: 200 μm. (c) CLSM images of core-shell MN array. Scale bar: 200 μm. (d, e) *Ex vivo* transdermal delivery of DiO-labled Fe-MSC-NVs in HA hydrogel and red-microspheres in HAMA hydrogel through the designed MN patch. CLSM images of (d) DiO-labled Fe-MSC-NVs, (e) red-microspheres after the MN patch applied on the rat skin. Scale bars: 200 μm. (f) Stimulated release profile of DiO-labled Fe-MSC-NVs in HA hydrogel and red-microspheres in HAMA hydrogel from the core-shell MN patch, respectively (n=3).

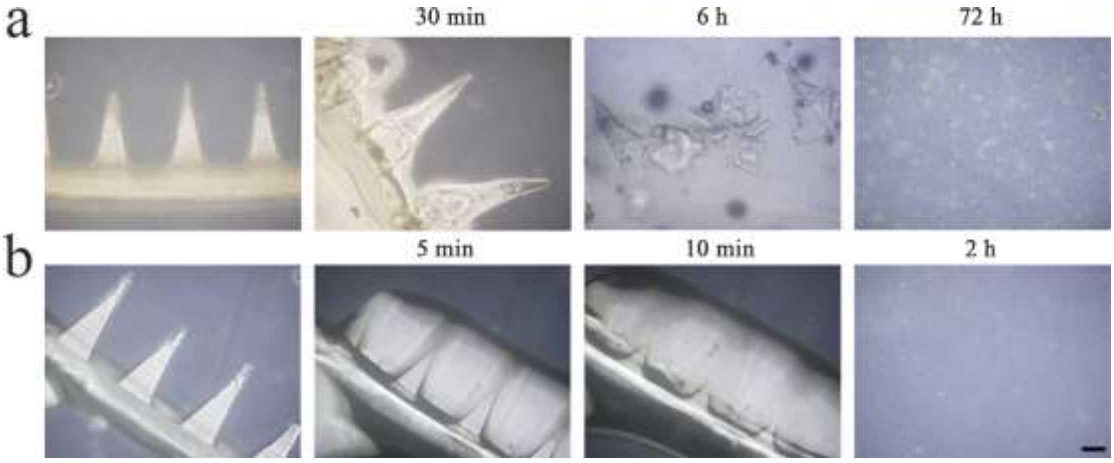

Figure S 6 The degradation of (a) HAMA/HA MN patch and (b) only HA MN patch in the PBS containing 100 units of hyaluronidase at 37°C at different times. Scale bar: 200 μm.

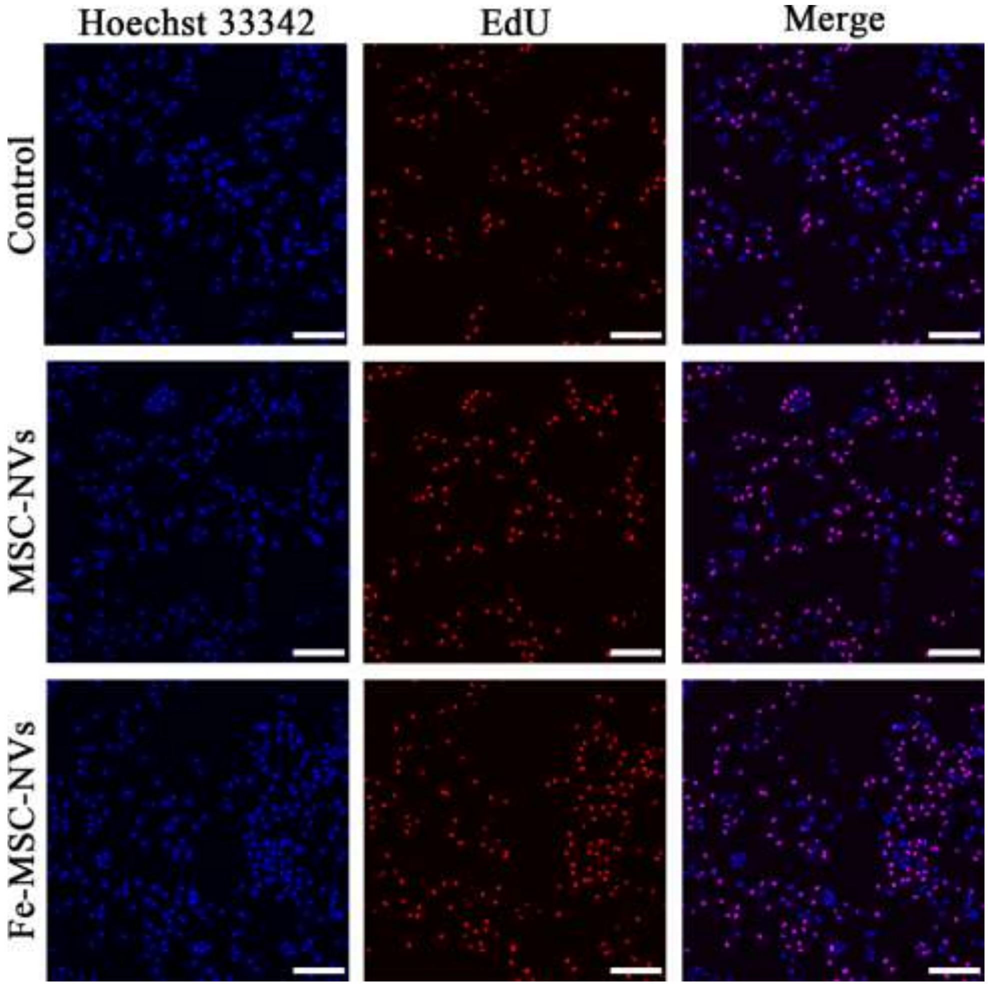

Figure S 7 Fluorescence images of HUVEC cultured in the medium supplementary with PBS (Control), MSC-NVs, or Fe-MSC-NVs for 24 h. Red: 5-Ethynyl-2'-deoxyuridine (EdU); blue: nucleus. Scale bars: 200μm.

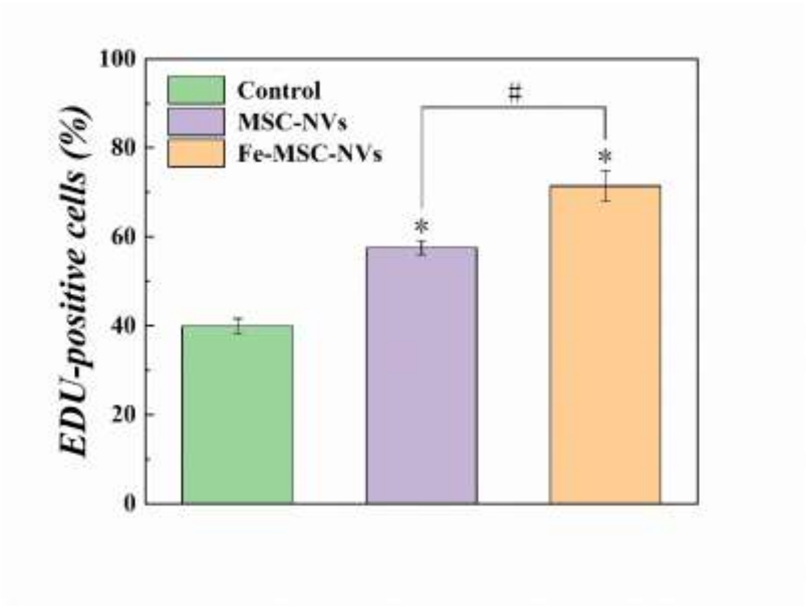

Figure S 8 Corresponding quantitative analysis of the proliferation assay in HUVEC.

n = 5, * $p < 0.05$ versus control. # $p < 0.05$.

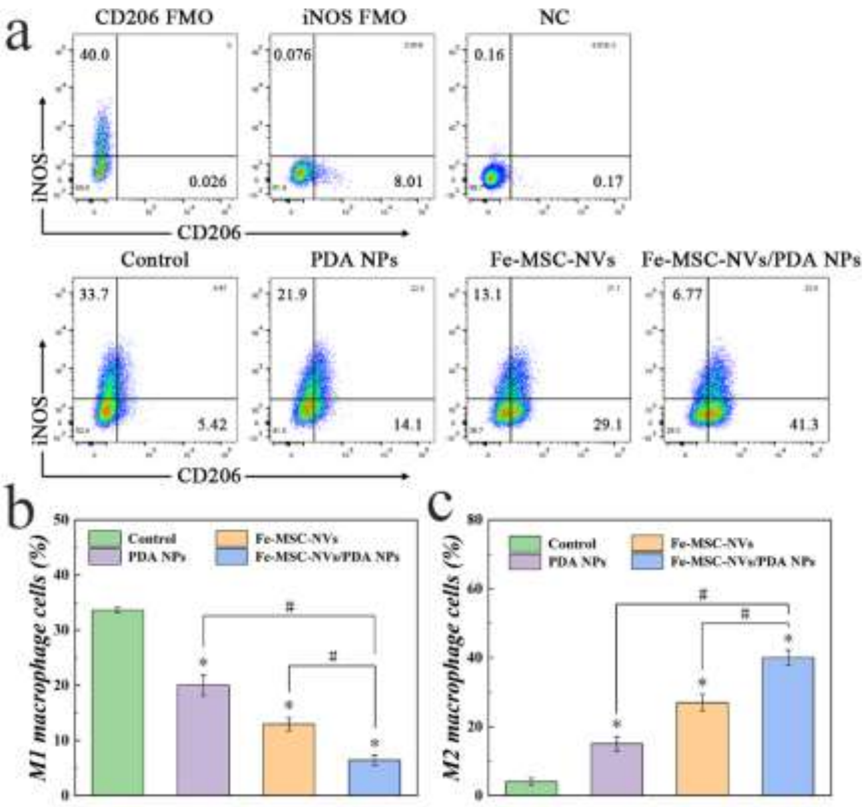

Figure S 9 (a) The CD206 FMO control and iNOS FMO control were used for gating different cell populations during FACS analysis; FACS analysis of iNOS and CD206 in F40/80$^+$CD11b$^+$ cells after being cocultured with PBS (negative control), LPS (control group), LPS/PDA NPs, LPS/Fe-MSC-NVs, or LPS/ Fe-MSC-NVs/PDA NPs. (b, c) Corresponding quantitative analysis of the positive expression percentage of (b) M1 macrophage cells (iNOS$^+$CD206$^-$) or (c) M2 macrophage cells (CD206$^+$iNOS$^-$) by flow cytometry. n = 5, * $p < 0.05$ versus control. # $p < 0.05$.

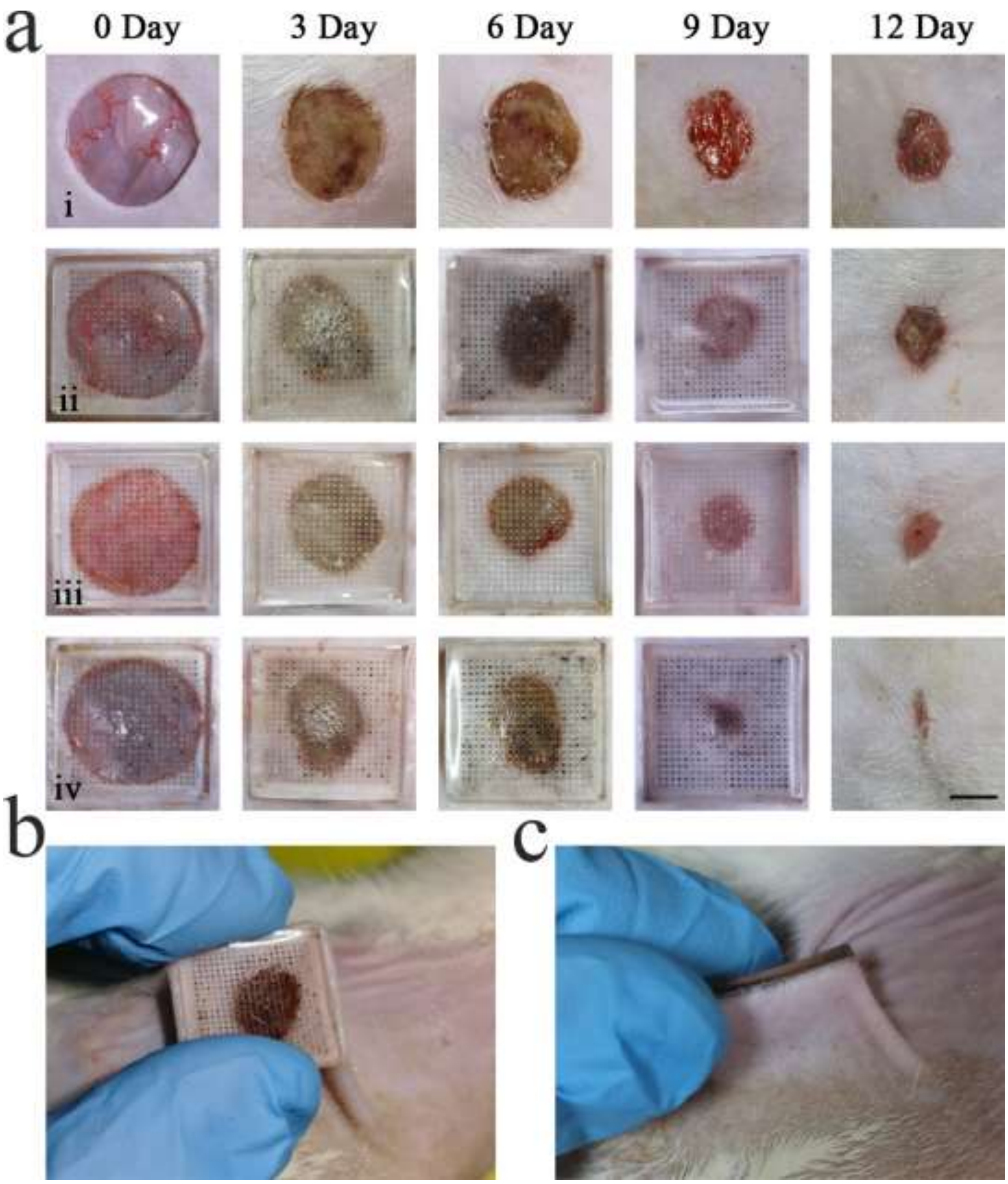

Figure S 10 (a) The photographic images of the patches applied on wound site in different treatment groups: control group, PDA MN, Fe-MSC-NVs MN, and Fe-MSC-NVs/PDA MN on days 0, 3, 6, 9, 12. Scale bar: 5 mm. The photographic images of the patches applied on wound site: (b) the front side and (c) the side view

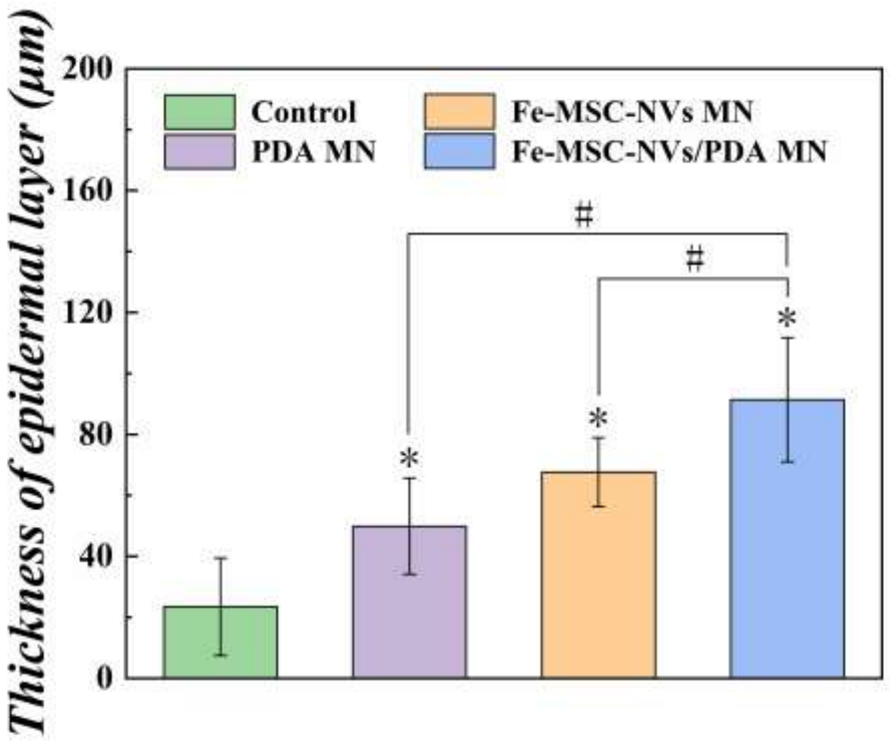

Figure S 11 Thickness analysis of epidermal layer in different treatment groups: control group, PDA MN, Fe-MSC-NVs MN, and Fe-MSC-NVs/PDA MN on day 12. n = 6, * $p < 0.05$ versus each control group. # $p < 0.05$.

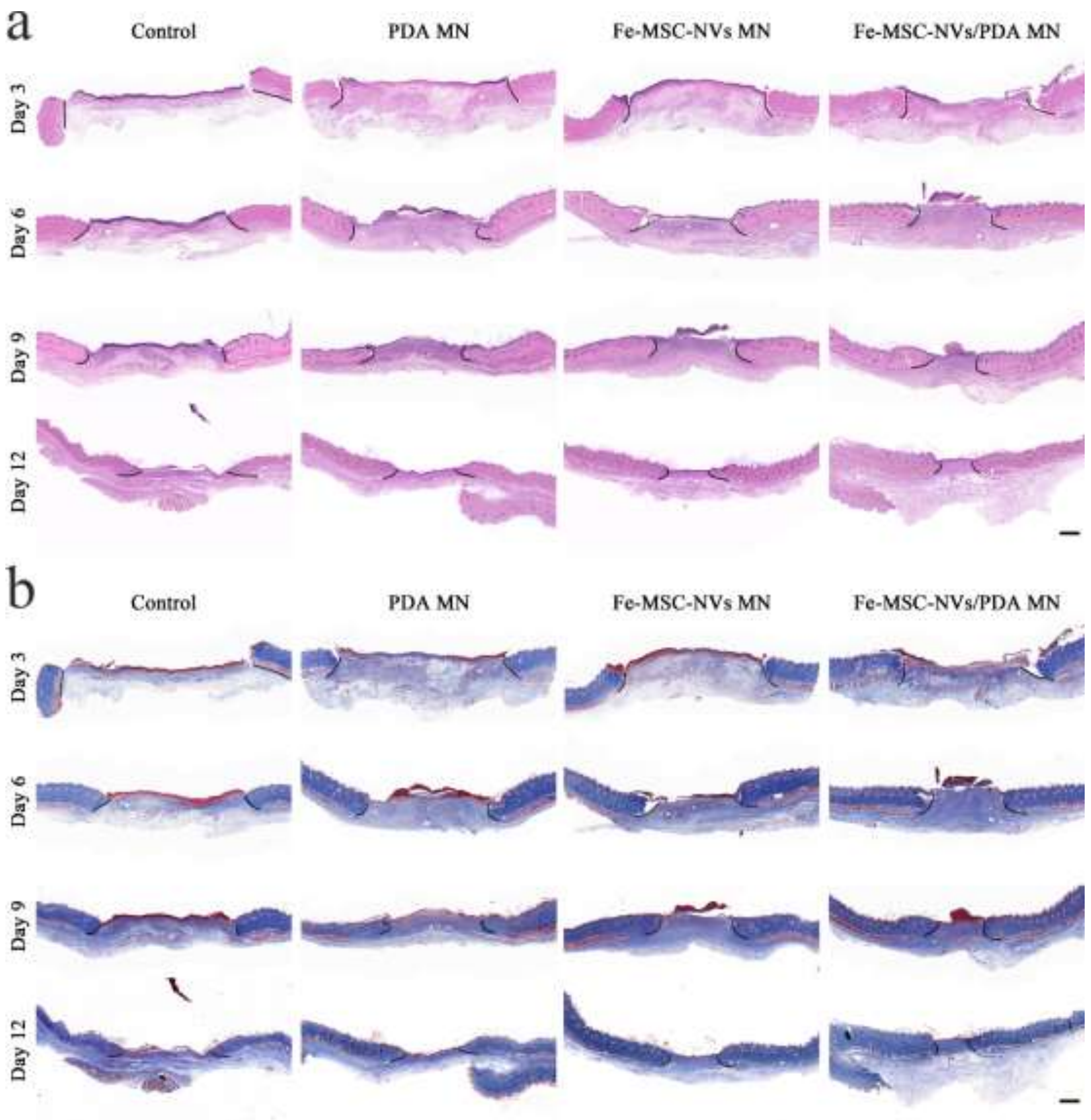

Figure S 12 Representative images of (a) hematoxylin and eosin (H&E) staining and (b) Masson's trichrome staining in different treatment groups: control group, PDA MN, Fe-MSC-NVs MN, and Fe-MSC-NVs/PDA MN on days 3, 6, 9, 12. Scale bars: 1 mm.

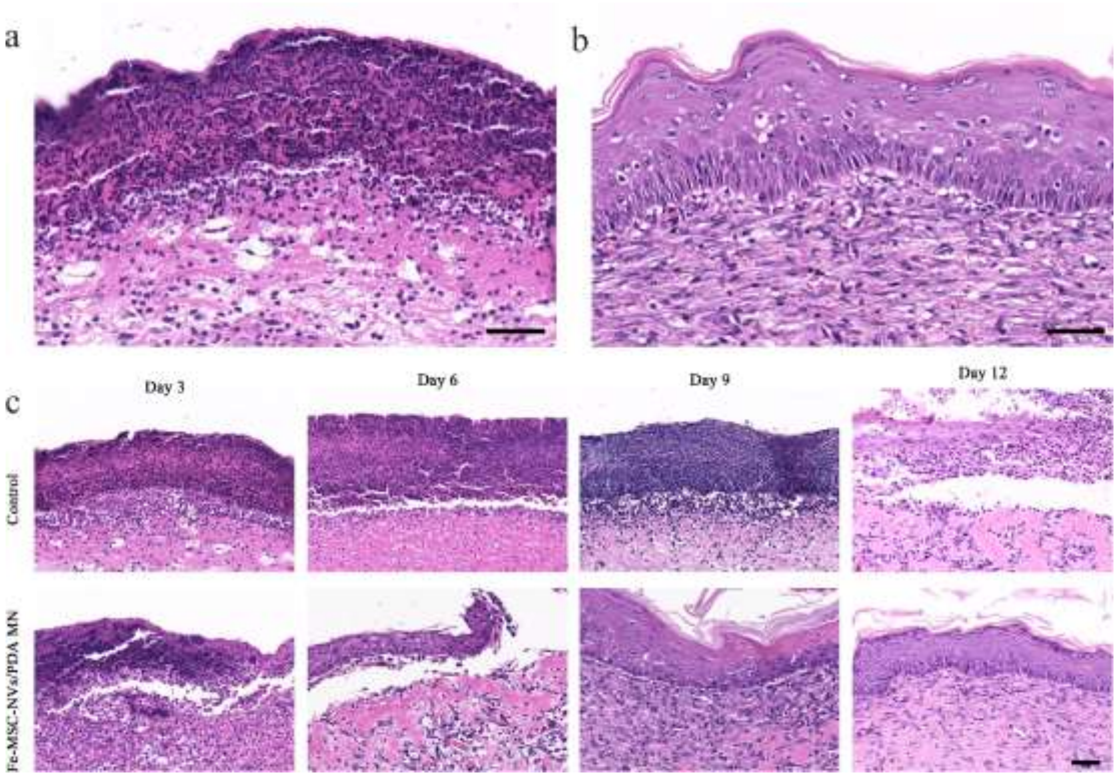

Figure S 13 Representative images of (a) wound crust and (b) epidermal layer in skin wounds. Scale bars: 50 µm. (c) Representative images of H&E staining in control group and Fe-MSC-NVs/PDA MN group. Scale bar: 50 µm.

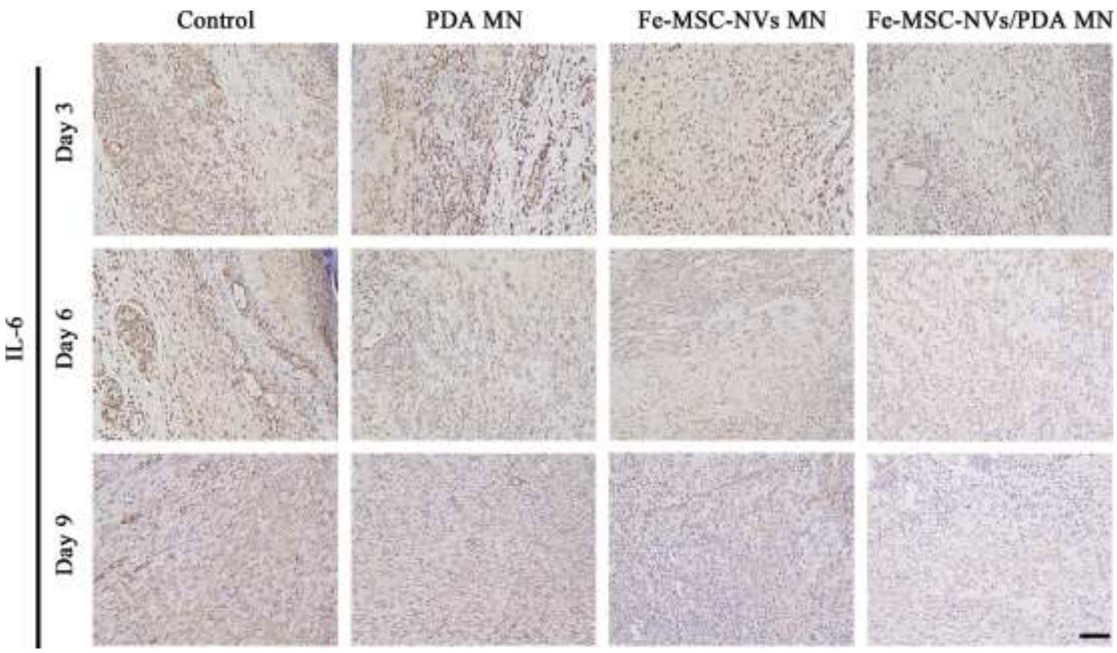

Figure S 14 Immunohistochemical staining of IL-6 of granulation tissues in different treatment groups: control group, PDA MN, Fe-MSC-NVs MN, and Fe-MSC-NVs/PDA MN on days 3, 6, 9. Scale bar: 100 μm.

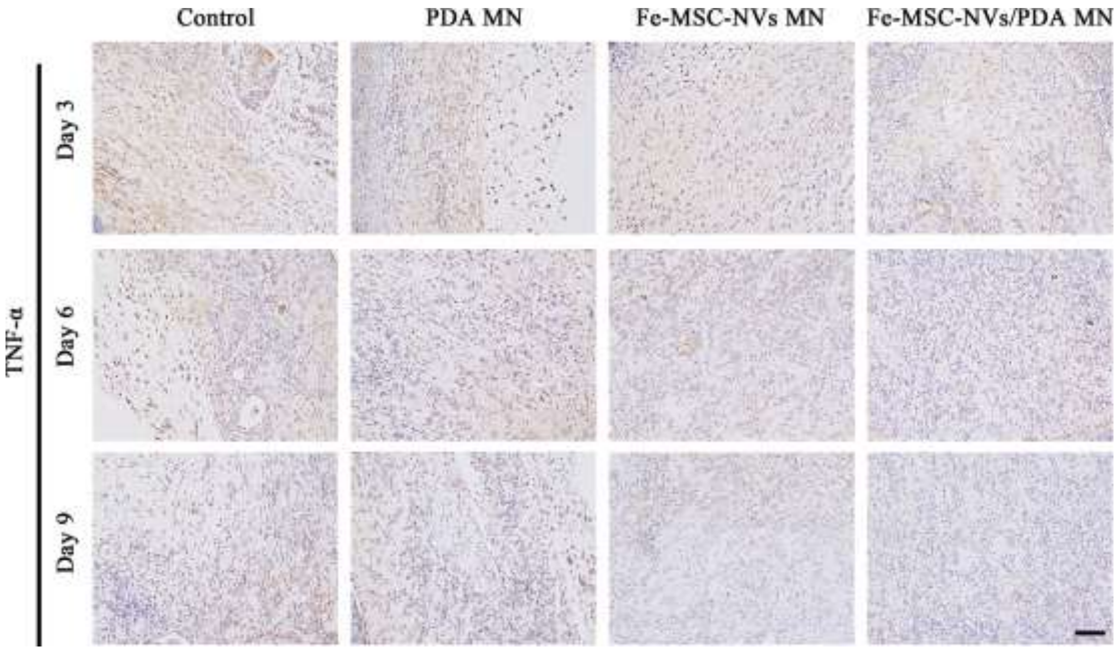

Figure S 15 Immunohistochemical staining of TNF-α of granulation tissues in different treatment groups: control group, PDA MN, Fe-MSC-NVs MN, and Fe-MSC-NVs/PDA MN on days 3, 6, 9. Scale bar: 100 μm.

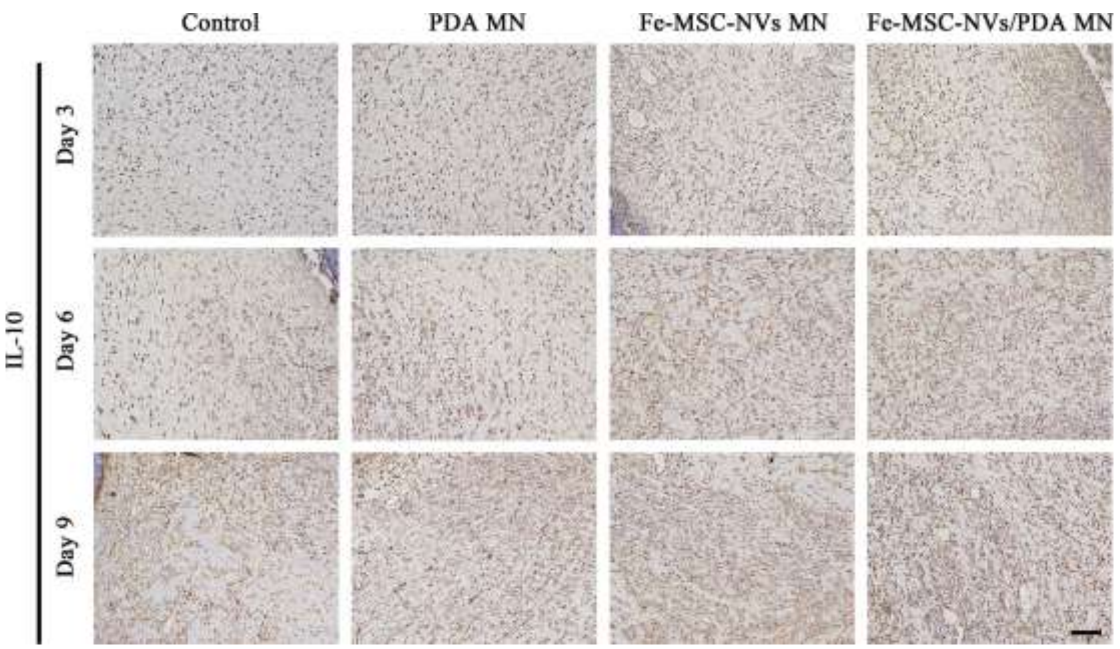

Figure S 16 Immunohistochemical staining of IL-10 of granulation tissues in different treatment groups: control group, PDA MN, Fe-MSC-NVs MN, and Fe-MSC-NVs/PDA MN on days 3, 6, 9. Scale bar: 100 μm.

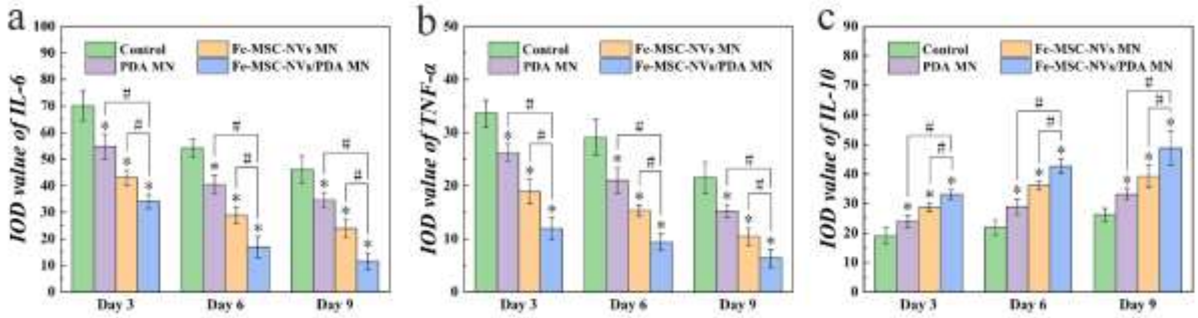

Figure S 17 Corresponding quantitative analysis of the mean value of the integrated optical density (IOD) for immunohistochemical staining of (a) IL-6, (b) TNF-α, and (c) IL-10 in different treatment groups. n = 6, * $p < 0.05$ versus control. # $p < 0.05$.

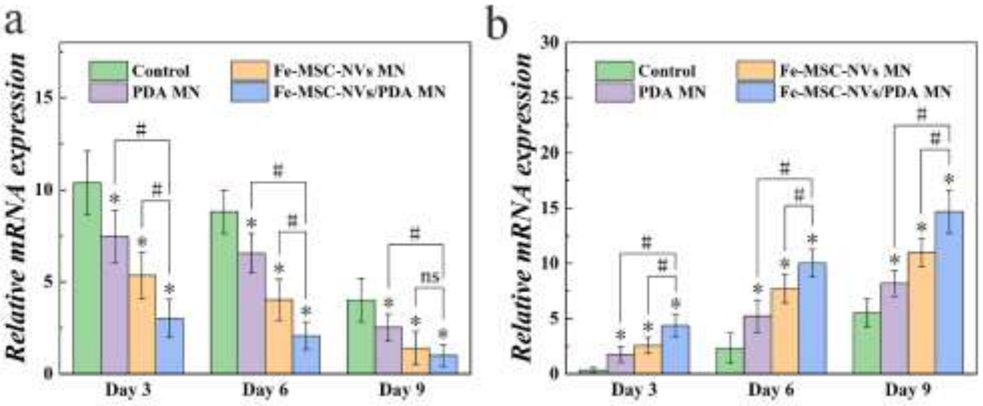

Figure S 18 Relative mRNA expression of (a) iNOS and (b) Arg-1 in granulation tissues of wounds in different treatment groups: control group, PDA MN, Fe-MSC-NVs MN, and Fe-MSC-NVs/PDA MN on days 3, 6, 9. n = 6, * $p < 0.05$ versus each control group. # $p < 0.05$.

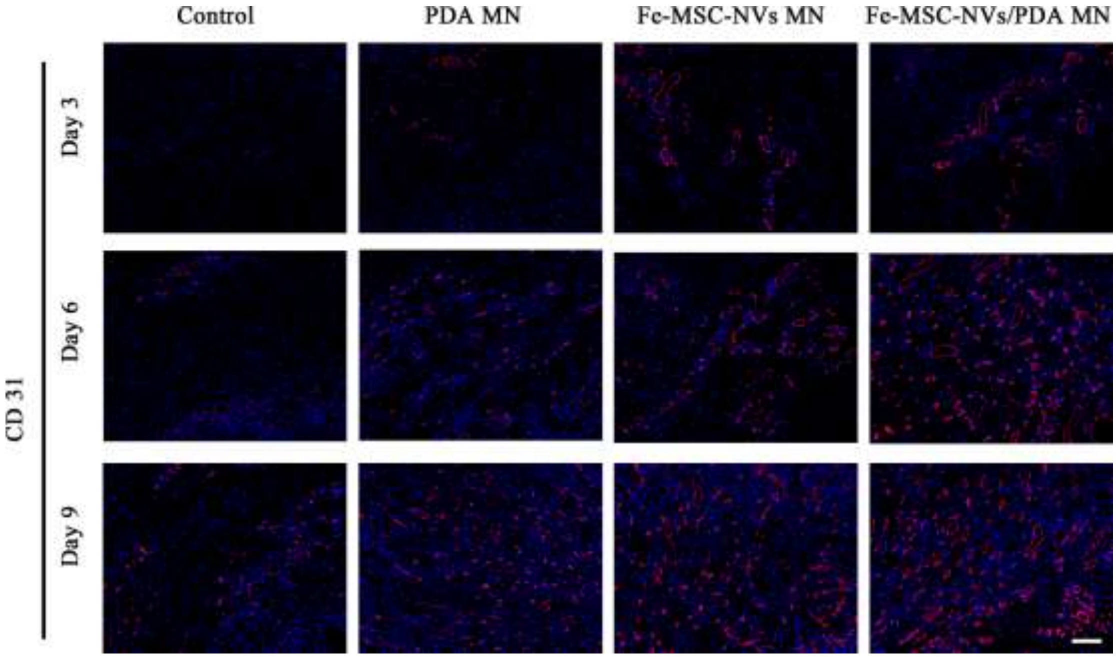

Figure S 19 Immunofluorescence staining of CD31 of granulation tissues in different treatment groups: control group, PDA MN, Fe-MSC-NVs MN, and Fe-MSC-NVs/PDA MN on days 3, 6, 9. Scale bar: 200 µm.

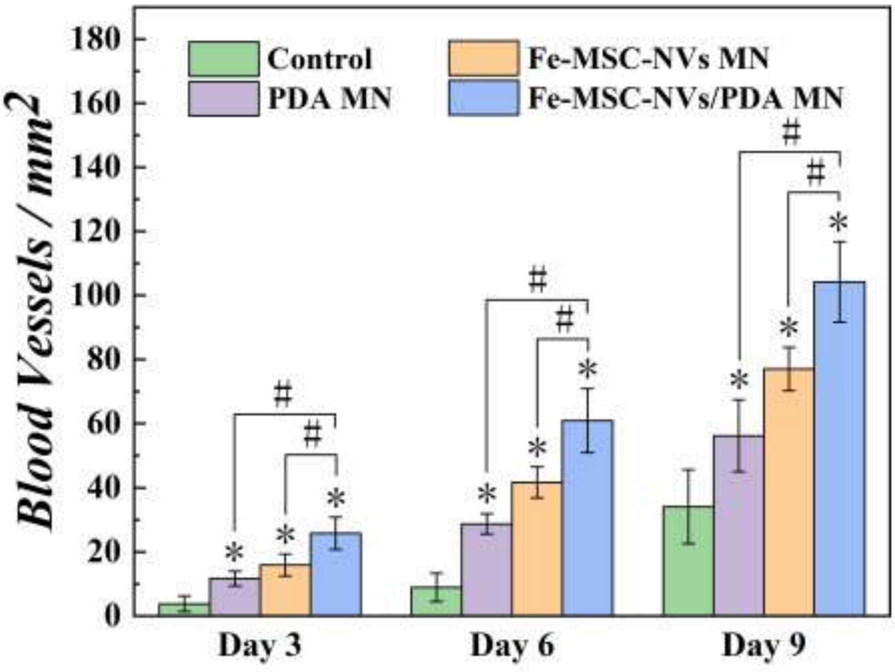

Figure S 20 Corresponding quantitative analysis of microvessel area stained by CD31 in different treatment groups on days 3, 6, 9. n = 6, * $p < 0.05$ versus control. # $p < 0.05$.

Table 1 Target gene primer sequences

Gene	Forward	Reverse
Human VEGF	TTGCCTTGCTGCTCTACCTC	AGCTGCGCTGATAGACATCC
Human HIF-1α	GTGGTGGTTACTCAGCACTTTT	CGTCCCTCAACCTCTCAGTT
Human HGF	GACGCAGCTACAAGGGAACA	AAAAGCTGTGTTCGTGTGGT

Human FGF2	CCACCTATAATTGGTCAAAGTGG	GAAACGAGGGAGAAAGGATGGA
Human Ang-1	GTGCTCACGTGGCTCGACTATA	GCACAGCAAGCTCAGCAGTT
Human TGFβ-3	TACTGCTTCCGGTGAGACTG	CAGGGACCCTCTGTTGAGTG
Human IL-6	GACAGCCACTCACCTCTTCA	CCTCTTTGCTGCTTTCACAC
Human GAPDH	ATGGGGAAGGTGAAGGTCG	GGGGTCATTGATGGCAACAATA
Rat iNOS	CCCTAAGAGTCACAAGCATC	AGGGTGTCGTGAAAAATCTC
Rat Arg-1	TGCCCTCTGTCTTTTAGGGCT	CTCCTCGAGGCTGTCCCTTA
Rat GAPDH	GCATCTTCTTGTGCAGTGCC	TACGGCCAAATCCGTTCACA
